# Efficacy of high-intensity interval and continuous endurance trainings on cecal microbiota metabolites and inflammatory factors in diabetic rats induced by high-fat diet

**DOI:** 10.1371/journal.pone.0301532

**Published:** 2024-04-16

**Authors:** Sogand Solouki, Sattar Gorgani-Firuzjaee, Hanieh Jafary, Maryam Delfan

**Affiliations:** 1 Department of Biology, Science and Research Branch, Islamic Azad University, Tehran, Iran; 2 Department of Medical Laboratory Sciences, School of Allied Health Medicine, AJA University of Medical Sciences, Tehran, Iran; 3 Clinical Biochemistry, School of Allied Medical Sciences, Infectious Diseases Research Center, AJA University of Medical Sciences, Tehran, Iran; 4 Department of Exercise Physiology, Faculty of Sport Sciences, Alzahra University, Tehran, Iran; University of Jeddah, SAUDI ARABIA

## Abstract

Physical exercise is known to modulate the intestinal microbiota composition and control the symptoms of metabolic syndrome. In this research, we intend to investigate and compare the effect of high-intensity interval and continuous endurance trainings (HIIT and CET) on cecal microbiota metabolites and inflammatory factors in diabetic rats. A number of Wistar rats were made diabetic by a high-fat diet and trained under two types of exercise protocols, HIIT and CET. After taking samples from the cecal tissue and serum of rats to reveal the effect of exercise, three microbial species from the *Firmicute* and *Bacteroid* phyla, which are the main types of intestinal microbes, and their metabolites include two short-chain fatty acids (SCFAs), butyrate and propionate and also, the inflammatory factors TLR4 and IL6 were analyzed through quantitative polymerase chain reaction (qPCR), high-performance liquid chromatography (HPLC), and Enzyme-linked immunosorbent assay (ELISA) methods. In general, exercise while increasing the representative of *Firmicute* has caused a relative reduction of Bacteroides and improved the concentration of SCFAs. In this regard, HIIT outperforms CET in up-regulating *Akkermansia* and *Butyrivibrio* expression, and butyrate and propionate metabolites concentration. Also, both exercises significantly reduced cecal expression of TLR4 and sera concentration of IL6 compared to the diabetic group, although the reduction rate was higher in the CET group than in HIIT. Our findings suggest that some symptoms of metabolic syndrome such as intestinal dysbiosis and the resulting metabolic disorders are better controlled by HIIT and inflammation by CET. Certainly, more extensive research on other contributing factors could help clarify the results.

## Introduction

Nowadays, due to the change in human lifestyle towards inactivity and increased consumption of high-fat foods, obesity is known as one of the effective clinical risk factors in the development of metabolic syndrome and insulin resistance [1, 2]. On the other hand, intestinal microbiota and its metabolites have attracted a lot of attention as one of the environmental factors involved in obesity management and diabetes prevention [3, 4]. About 90% of the intestinal microbial population consists of two families: *Firmicutes* and *Bacteroidetes* [5]. Recent studies have shown that the compositional and functional changes of the intestinal microbiota through several mechanisms, including increased intestinal permeability and low-grade endotoxemia, changes in the production of short-chain fatty acids (SCFAs) and branched-chain amino acids (BCAAs), and disturbances in the metabolism of bile acids play an important role in the development of metabolic syndrome diseases such as cardiovascular, type 2 diabetes, obesity, and intestinal inflammation [6–8]. Disturbance in the intestinal microbiome causes more absorption of monosaccharides, an increase in the level of lipogenic enzymes, and the occurrence of insulin resistance [9, 10]. Evidence shows that the development of obesity, diabetes, and pre-diabetes are associated with changes in the gut microbiota [11, 12]. Intestinal dysbiosis changes the amount of metabolites resulting from their fermentation [13–15]. For example, a change in the intestinal microbiota causes a change in the production of SCFAs including acetate, butyrate, propionate, valeric acid, succinate and caproic acid and enzymes that directly affect the regulation of glucose and insulin and ultimately contribute to the development of obesity.

SCFAs are the main and final products of the fermentation process resulting from the activity of anaerobic bacteria in the large intestine [16]. Acetic, propionic, and butyric acids make up 95% of the total content of SCFAs produced in the human colon [17]. They play an essential role in the proper functioning of the digestive system. For example, butyric acid has local anti-inflammatory, immune system regulating, and local anti-neoplastic effects. In addition to their roles as energy substrates, immune cell regulators, and gut hormone modulators, SCFAs have been shown to prevent obesity and insulin resistance and induce thermogenesis in brown fat [14, 16, 17].

Gut microbiota and its mucosal integrity are influenced by diet, environment, and other lifestyle factors, including physical activity [18]. On the other hand, several studies show that there is a strong relationship between obesity and inactivity, which directly causes insulin resistance and increases the risk of metabolic syndrome [19–21]. Considering the existence of a possible link between obesity and changes in the gut microbiome, it is important to know what factors are involved in intestinal microbial changes, and how their correction can help to eliminate obesity and prevent metabolic syndrome.

Performing regular sports activities has a modulating effect on the intestinal microbiota [22–25]. It is important to know how different types of exercise interventions (different intensities and periods) affect the content and metabolites of intestinal microbes and the control of diabetes and inflammation. For this purpose, in this study, the effect of two major categories of sports, including high-intensity interval training (HIIT) and continuous endurance training (CET) on the gene expression of the bacteria *Butyrivibrio fibrisolvens* and *Ackermans Muciniphila* from the family of *Firmicutes* and *prevotella copri* from the family of *Bacteroidetes* is investigated by quantitative polymerase chain reaction (qPCR) method. Also, we analyze the change in the concentration of SCFAs caused by the activity of *firmicutes* including butyrate and propionate by high-performance liquid chromatography (HPLC) method. Finally, changes in sera concentration of Interleukin 6 (IL6) inflammatory factor and cecal gene expression of Toll-like receptor 4 (TLR4) are measured by Enzyme-linked immunosorbent assay (ELISA) and qPCR methods, respectively. Due to being in the upstream of the inflammatory cascade, these biomarkers play an important role in mediating the inflammatory response and activating other cytokines [26–28]. They also play an essential role in regulating intestinal permeability and maintaining the integrity of the epithelial barrier [29–31].

## Materials and methods

### Study subjects and ethical considerations

**Fig 1:**
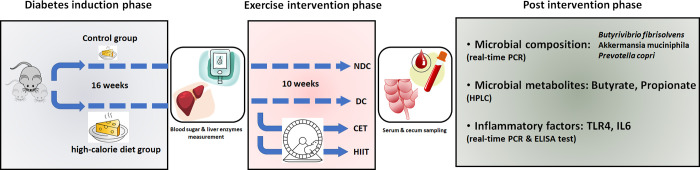
General experiment phases. The experiment is conducted in three stages, including 1) induction of diabetes by a high-fat diet, 2) exercise intervention with HIIT and CET protocols, and 3) measuring the effect of the intervention on the microbiome and metabolites of the cecum and serum inflammatory factors. NDC, non-diabetic control; DC, diabetic control; CET, continuous endurance training; HIIT, high-intensity interval training. The symbolic items in the figure drawn by the authors are for illustrative purposes only.

The study is conducted on 45 male Wistar rats (purchased from Razi Institute, Iran) with an average weight of 200±10 g and an age of 8 weeks; Animals were housed individually in cages in a room that was controlled for temperature (22°C), humidity and light (12 h light: dark cycle). The animal housing was well ventilated. All rats had free access to laboratory chow and tap water ad libitum. Food and water containers were sterilized daily. The health status, activity and eating of the rats were monitored daily. The animal food was dry and high in fat, and to prevent it from spoiling, it was kept in a freezer at -18 degrees, and the rest was kept in the refrigerator while it was being used. The water, food and bedding of the animals have been changed every other day. All animal treatments were considered humanly and in compliance with the recommendation of the animal care committee of AJA University of Medical Sciences and the principles of laboratory animal care (Approval Code: IR.AJA UMS REC 1400.287) (Fig 1). Before starting the experiment, the rats were given enough time to familiarize themselves with the environment and training conditions. After one week of acclimatization to the environment, the rats were divided into two groups, control and under a 16-week high-calorie diet. At the end of the 16th week, two rats from the control group and four rats from the diabetes group (i.e., high-calorie diet) were randomly selected, and their fasting blood sugar and insulin resistance (HOMA-IR) were checked. Blood sugar above 250 mg/dL (13.5 mM/L) and HOMA > 3 were considered as criteria to confirm diabetes. Diabetic rats did not receive any insulin treatment during the experiment. Next, the rats were randomly divided into four groups of 8, including 1) non-diabetic control (NDC), 2) diabetic control (DC), 3) continuous endurance training (CET), and 4) high-intensity interval training (HIIT) (13 rats were excluded from the study: 5 diabetic rats were anesthetized and euthanized immediately after leg injury during exercise so as not to suffer from diabetic wound pain, and 8 rats were excluded due to not reaching the diabetes threshold). After 24 h of the last exercise session and after an overnight fasting, the rats were taken one by one to the dissection room and anesthetized by intra peritoneal injection of ketamine (90 mg per kg of body weight) and xylazine (10 mg per kg of body weight). After anesthetization, we employed the widely recognized laboratory procedure of cardiac puncture to efficiently extract the entire blood volume from the left ventricle of the animals, a process conducted in the dorsal recumbency position, ultimately leading to the humane euthanasia of the subject [32, 33]. Next, the animals were dissected and the tissues of the cecum were kept in liquid nitrogen for further study. The serum samples were stored at -80 degrees. Disposable surgical blade and autoclaved surgical set have been used for dissecting. The rest of the animal carcasses were collected in special infectious garbage bags and bins and delivered to the incinerator department for disposal. All procedures were carried out under the supervision of animal care experts and the ethics committee of AJA University of Medical Sciences and based on the latest national guidelines for animal care [34].

### Diet and exercise protocol

The high-calorie diet was purchased from Razi Institute with the formulation of 30% animal fat, 25% fructose and 45% basic animal food. Rats were familiarized with the exercise protocol one week after the induction of diabetes with five weekly training sessions. Before the start of the first session, the rats were trained at a slower speed than the defined pattern. After that, the main training was done for nine weeks.

The exercise program is as follows: The diabetic (DC) and non-diabetic (NDC) control groups did not participate in any exercise program, but to create the same conditions as the other groups, they were placed on a stationary treadmill for 10 to 15 minutes five times a week. CET and HIIT groups performed the protocol in Table 1 for ten weeks.

**Table 1:** CET and HIIT exercise protocol.

Practice procedures	Warming up	Context of exercise protocol			Cooling down
Practice time (min)	5 min	30 min (CET)			5 min
		**1 min** (4 HIIT repeats)	**3 min** (HIIT recovery)		
Intensity of practice (% of VO2 max)	30–40%	50–60% (CET)			30–40%
		**30–35%** (4 HIIT repeats)		**85–90%** (HIIT recovery)	
Decline	0	0			0

VO2 max, maximal oxygen consumption or maximal aerobic capacity; CET, continuous endurance training; HIIT, high-intensity interval training.

### Sample collection

Venous blood samples to measure fasting blood sugar, insulin resistance (Table 2), and serum concentration of inflammatory marker IL6 were stored in tubes without anticoagulant and centrifuged at 4000 x g for 10 minutes. Finally, the isolated serum was stored at -80°C until the experiment started. Cecal tissue samples were removed from rats’ abdominal cavity immediately after euthanizing them and were stored in sterile microtubes at -80°C until the beginning of the instrumental analysis.

**Table 2:** Blood sugar analysis of the rats’ sera.

	NDC	DC	CET	HIIT
	Mean ± SE	Mean ± SE*	Mean ± SE^#^	Mean ± SE^
**FBS (mg/dL)**	119±22.57	305. 5±25.24 p<0.0001	199.12±40.28 p<0.0001	145.71±44.59 p<0.0001
**HOMA-IR (%)**	1.24±0.13	6.45±0.19 p<0.0001	2.78±0.2 p<0.0001	1.90±0.32 p<0.05

**Notes:** The p-values in

(*), (#), and (^) columns correspond to the intergroup comparison of NDC-DC, DC-CET, and DC-HIIT, respectively (n = 8, significance level: p ≤ 0.05, p ≤ 0.0001)

**Abbreviations**: NDC, non-diabetic control; DC, diabetic control; CET, continuous endurance training; HIIT, high-intensity interval training.

### Analysis of inflammatory factors IL6 and TLR4

#### Measurement of serum concentration of inflammatory marker IL6

Sera concentration of IL6 was measured by non-competitive solid-phase sandwich immunoassay (ELISA) using diagnostic kit of Karmania Parsgene Co., Iran, under the Cat. No. REF KPG-RIL6K. Based on this, the solid phase antibody captures the serum antigen and the standards and causes the antigen to be fixed by the unlabeled antibody in the solid phase. Washing removes unbound samples. Then the labeled antibody is added to react with the antigen and form a link in the solid phase. The unbound labeled antibody is removed by washing, and the remaining labeled antibody is measured. The intensity of the color is directly proportional to the concentration of IL6 in the serum. The optical absorbance of the samples was measured with an ELISA reader (Hyperion, Inc., FL, USA), at a wavelength of 450 nm. The standard curve was also formed by a 4-parameter algorithm.

#### Measurement of TLR4 gene expression in rat cecal tissue

The relative expression of TLR4 gene was measured by real-time PCR. Total cecal tissue RNA was extracted using a Karmania Parsgene RNA extraction kit under Cat. No. REF KPG-RNEK according to the relevant protocol. Total RNA was reverse transcribed using an Eppendorf gradient mastercycler (Hamburg, Germany) according to the instructions of the Karmania Parsegen cDNA synthesis kit (Tehran, Iran). To perform real-time PCR tests according to MIQE instructions [35], melted cDNA samples, primers designed by Pishgam Biotechnology Co. (Iran), SYBER Green Master Mix prepared by Anacel Co. (Iran), and ABI 7500 thermocycler with software version 2.3 were used (The primers sequences are listed in S1 Table [36, 37]). PCR temperature cycling protocol was set as follows and ran twice for each sample: Holing at 95°C for 15 min, Denaturation at 95°C for 20 sec, Annealing at 60°C for 60 sec, and Extension at 95°C for 30 sec. The amount of TLR4 gene expression in each sample was obtained using cycle threshold values through ABI 7500 (version 2.3). To normalize the data, TLR4 gene expression was compared with GAPDH, and the relative changes were calculated using the 2-ΔΔCT method.

### Quantification of target cecal bacteria by qPCR

Primers were selected to recognize *Prevotella copri*, *Akkermansia muciniphila*, and *Butyrivibrio fibrisolvens* species from the *Bacteroides* and *Firmicutes* phyla. DNA of the target bacteria was extracted from 200 mg of cecal tissue using a Karmania Parsgene extraction kit under KPG-DNKtb Cat. No. In the following, the ABI 7500 device (version 2.3) was used to quantify the genetic material. The necessary primers for real-time PCR were purchased from Pishgam Biotechnology Co., Iran (S2 Table [38–40]). Each 20 μl qPCR reaction solution consists of 10 μl SYBR Green Master Mix, 1 μl forward and reverse primer mix, 8 μl sterile distilled water, and 1 μl extracted DNA. For negative control, 1 μl of sterile distilled water was added to the reaction solution instead of template DNA. The PCR temperature cycle was set as TLR4, and the samples were analyzed twice. The gene expression level of the target bacteria was obtained based on CT values. The difference in gene expression of the target and Universal bacterium was normalized by the 2-ΔΔCT method.

### Analysis of short chain fatty acids butyrate and propionate in rat cecum

Butyrate and propionate were measured as secondary markers of exercise-induced changes in the gut microbiome via HPLC [41]. For this purpose, an Agilent 1200 HPLC-UV device (Santa Clara, USA) including a P400 quadruple gradient pump with a G1322A degassing vacuum, a manual sampler, and a G1314B detector at a wavelength of 210 nm was used. Chromatographic separation was performed on an Eclipse XDB-C18 column (150 mm x 4.6 mm) with a particle size of 5 μm. Data recording and processing were done with the chemstation program.

The liquid phase consists of a) a mixture of sodium monophosphate and deionized water with a pH of 2.2 adjusted by phosphoric acid and b) acetonitrile. Washing is done by gradient method. Working solutions with concentrations of 0.5 M and 0.05 M and HPLC-grade water were used to make calibrators (S3 Table). Finally, by adding HPLC-grade water to each tube, we brought the final volume to 1000 μl and vortexed them. We considered one of the concentrations made from the calibrators as a control sample. The blank sample is the same water used for HPLC. We used the liquid-liquid extraction method to prepare the samples. For this purpose, 1000 μl of HPLC water was added to 200 mg of each cecum tissue homogenized sample and vortexed. Then 100 μl of 37% HCL was added, and the samples were vortexed for 15 seconds. For extraction, 5 ml diethyl ether was added to each sample; the mixtures were gently rolled for 20 minutes, and centrifuged at 3500 rpm for 5 minutes. Then the supernatant obtained from each sample was transferred to sterile Falcons. 500 μl of NaOH solution with a concentration of 1 M was added to each sample and the mixtures were gently stirred for 20 minutes and then centrifuged at 3500 rpm for 5 minutes. The liquid phase was transferred to sterile tubes and 100 μl of 37% HCL was added to each sample and vortexed. In the last step, 10 μl of the samples were injected into the HPLC device. The schedule of passing the liquid phase through the column is specified in S4 Table.

### Statistical analysis

Experimental data were recorded in four groups of 8 members, including NDC, DC, CET, and HIIT, and were statistically analyzed by SPSS version 22 (IBM, New York, USA) and MATLAB version R2020b (MathWorks, Natick, MA, USA). The chi-square test was used to check the normality of data distribution. In the following, one-way analysis of variance (ANOVA) was used to examine the difference between the intervention and control group averages. After confirming the hypothesis that there is a difference between the test group averages, the Tukey-Kramer method of multiple comparisons was used to determine the level of difference between the exercise groups and the diabetic and non-diabetic control groups. Statistical significance levels from maximum to minimum were considered as 0.001, 0.01, and 0.05 respectively. The statistical variables of each group, including mean, median, standard deviation (STD), standard error (SE), and variation span were calculated and announced in the tables and graphs of the results. Notably, data outside 1.5 times the interquartile range were recognized as outliers and excluded from the statistical analysis process. A linear regression model with a correlation coefficient of 0.99 was used to draw the standard curve of chromatography and ELISA tests.

## Results

### The effect of exercise interventions on inflammatory factors IL6 and TLR4

**Fig 2:**
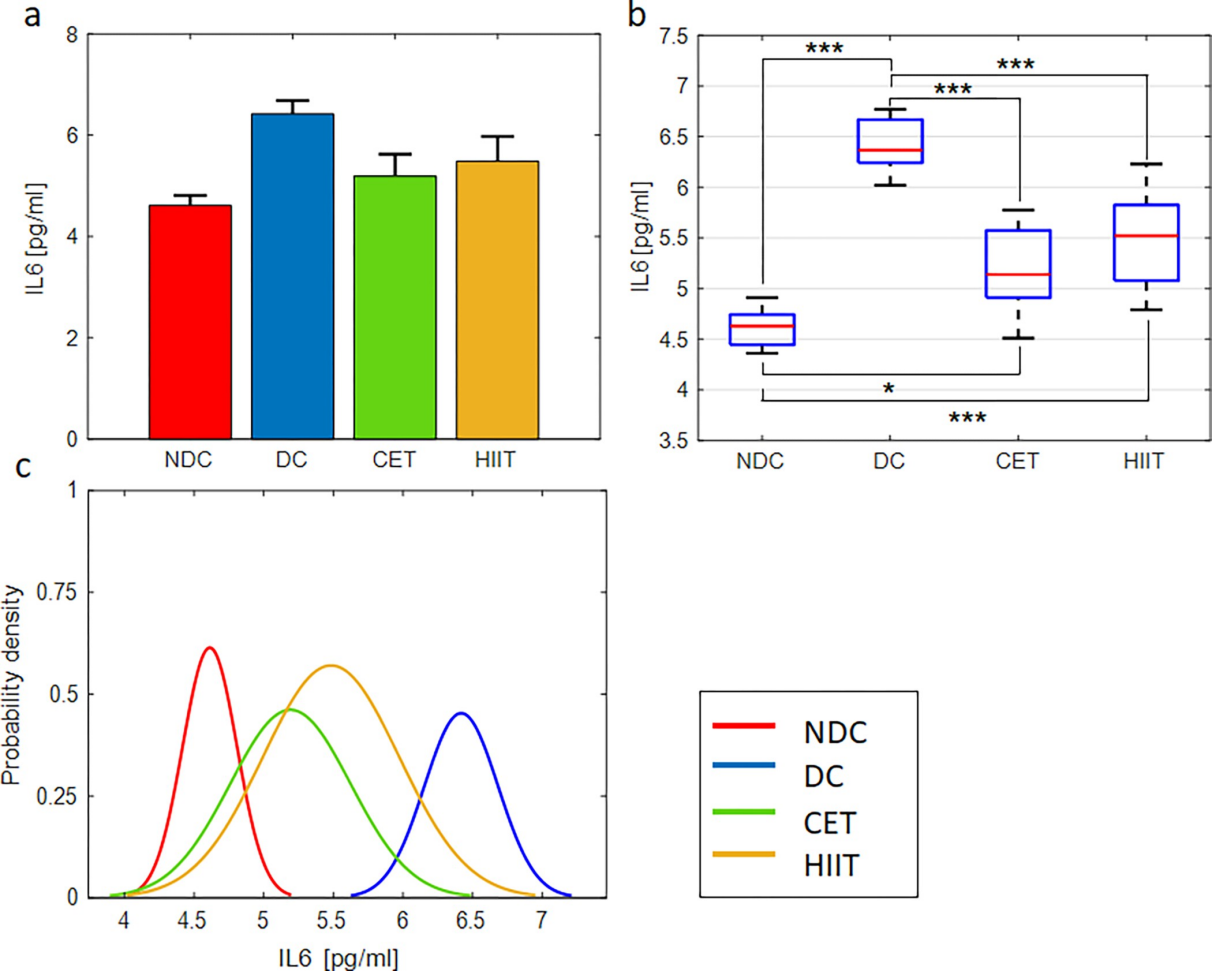
Exercise intervention effect on sera concentration of IL6. (a,b) bar and box plots representing the relative value and variation span of IL6 concentration in NDC, DC, CET, and HIIT groups. Medians in (b) are marked with red lines. Tukey’s multiple comparison test is used to determine the difference level between the exercise and control groups, ***p ≤ 0.001, **p ≤ 0.01, and *p ≤ 0.05, n = 8. (c) Probability density function representing normal distribution of IL6 concentration in the studied groups.

**Fig 3:**
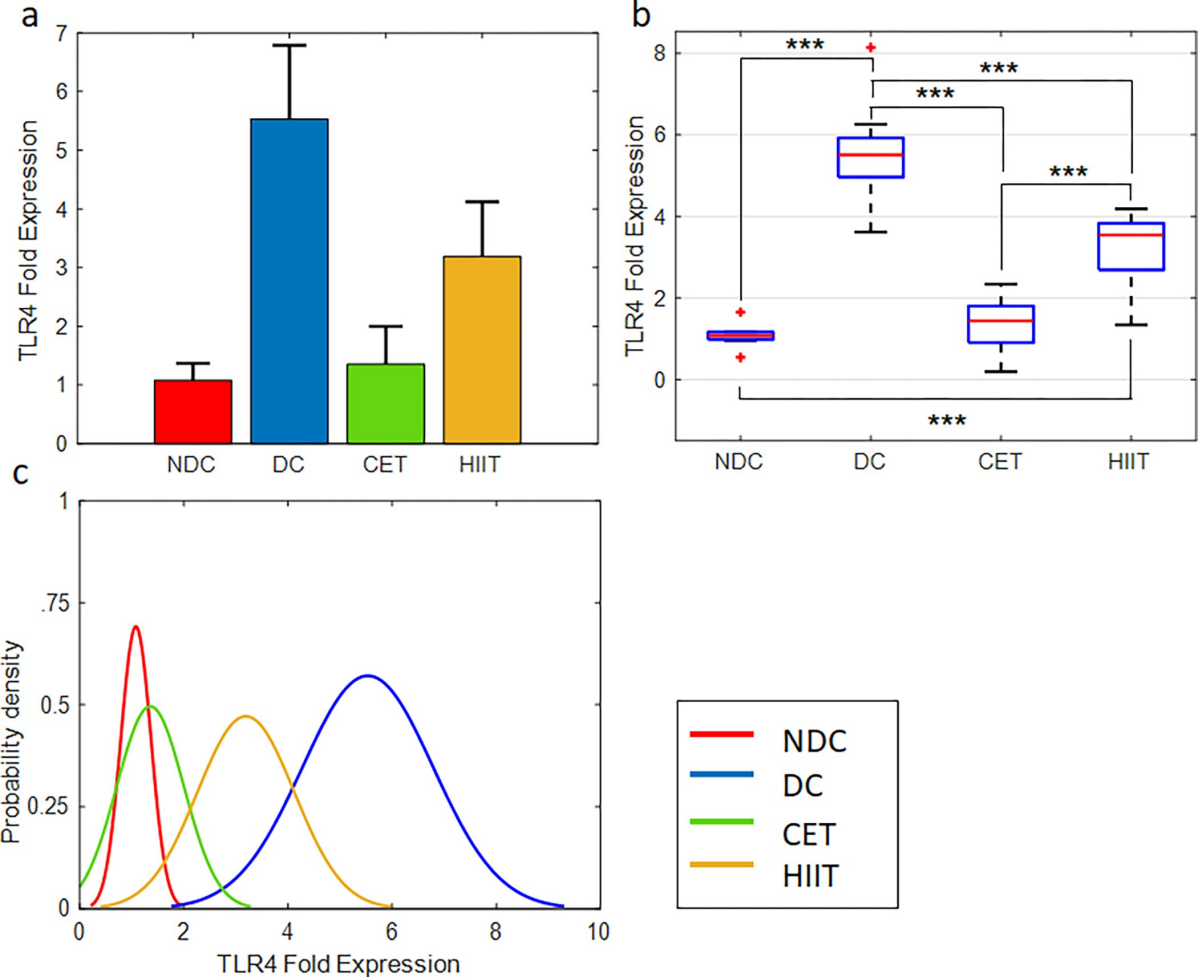
Exercise intervention effect on TLR4 expression in cecal tissue. (a,b) bar and box plots representing the relative value and variation span of TLR4 expression in NDC, DC, CET, and HIIT groups. Median and outlier data in (b) are marked with red line and star, respectively. Tukey’s multiple comparison test is used to determine the difference level between the exercise and control groups, ***p ≤ 0.001, **p ≤ 0.01, and *p ≤ 0.05, n = 8. The normalized relative gene expression values are obtained based on 2-ΔΔCT method, comparing TLR4 with GAPDH. (c) Probability density function representing normal distribution of TLR4 gene expression in the studied groups.

The effect of exercise interventions and diabetes on the inflammatory factors IL6 and TLR4 measured by ELISA and qPCR methods, respectively, are shown in Figs 2 and 3 and S5 and S7 Tables. In accordance with previous findings [42–46], diabetes has led to a significant increase in IL6 concentration in serum and TLR4 gene expression in cecal tissue samples. Comparing the diabetic control group with CET and HIIT confirms the positive effect of exercise on reducing the level of inflammatory factors. In other words, both exercise interventions, regardless of their type, have reduced the level of inflammation. But the reduction rate in CET is slightly higher than HIIT, which can be a sign that this exercise is more effective on inflammatory factors. As shown in Fig 3B, the level of the inflammatory factor TLR4 in the CET exercise group is significantly lower than that of the HIIT group, so the expression of this inflammatory factor has been successfully reduced to its normal level in the non-diabetic control (NDC) group. However, the level of IL6 concentration (Fig 2B) in the CET group is still significantly different from the NDC group. The P-value obtained from the one-way ANOVA confirms the hypothesis of the effectiveness of exercise interventions on the inflammatory factors IL6 and TLR4 (S6 and S8 Tables). Also, the results of the multiple comparisons between the four groups of NDC, diabetic control (DC), CET, and HIIT are shown in S5 and S7 Tables. Column 6 of these tables shows the significant level of difference between the groups in pairs. The fourth column shows the difference between the averages of the compared groups. The third and fifth columns show the lower and upper limits of the 95% confidence interval for the mean difference of the compared groups. In the lower part of the aforementioned tables, the average, standard deviation (STD), and standard error (SE) of all four groups are announced separately. The probability density function of the studied groups is also shown in Figs 2C and 3C. As can be seen, all groups follow the normal distribution pattern, which is a prerequisite for the ANOVA test. In addition, the distribution of exercise groups is in a range between diabetic and non-diabetic groups (tending to the non-diabetic group). This indicates the decreasing trend of inflammatory factors in the exercise groups compared to the diabetic group.

### The effect of exercise interventions on intestinal microbial content

**Fig 4:**
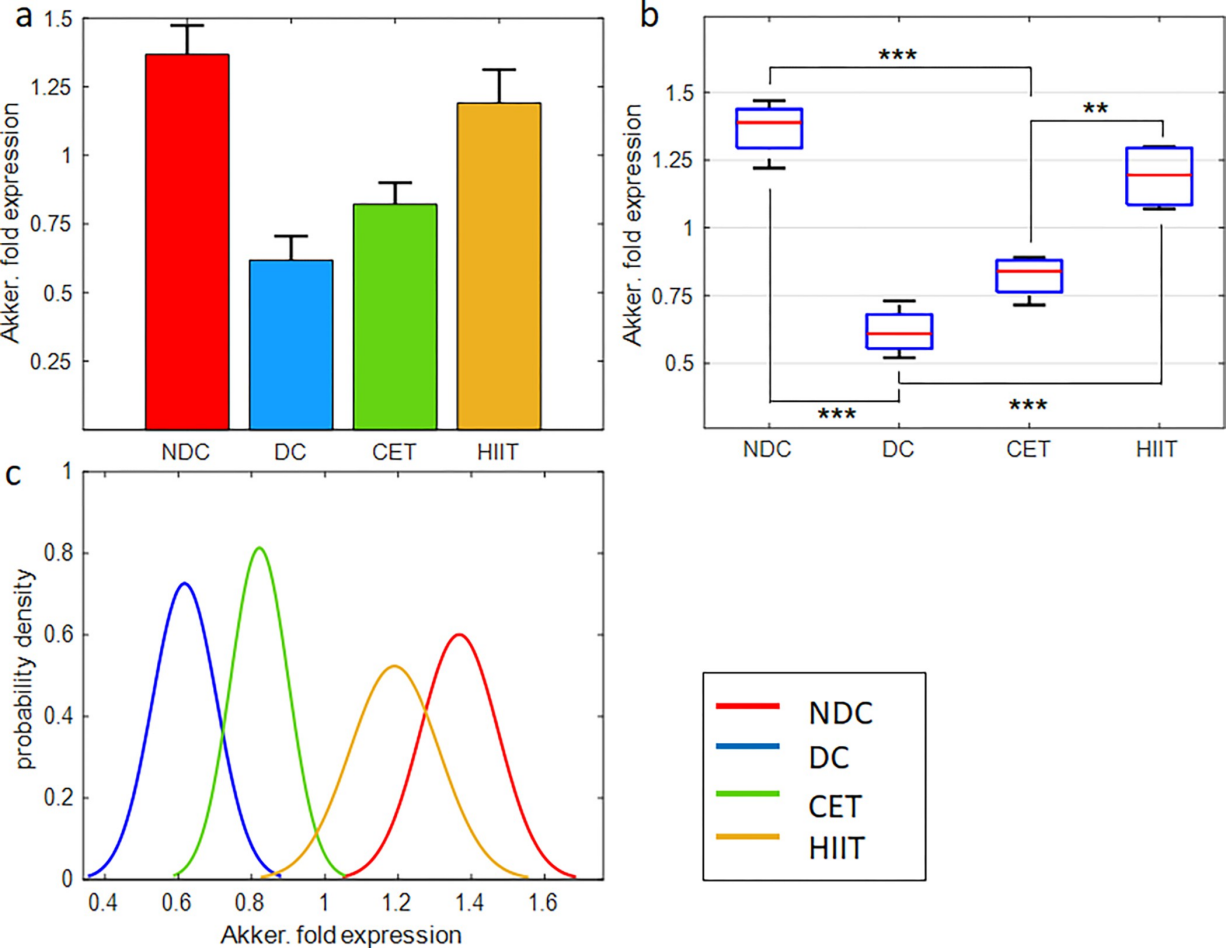
Exercise intervention effect on *Akkermansia muciniphila* expression in cecal tissue. (a,b) bar and box plots representing the relative value and variation span of *Akkermansia* expression in NDC, DC, CET, and HIIT groups. Medians in (b) are marked with red lines. Tukey’s multiple comparison test is used to determine the difference level between the exercise and control groups, ***p ≤ 0.001, **p ≤ 0.01, and *p ≤ 0.05, n = 8. The normalized relative gene expression values are obtained based on 2-ΔΔCT method, comparing target with universal bacterium. (c) Probability density function representing normal distribution of *Akkermansia* gene expression in the studied groups.

**Fig 5:**
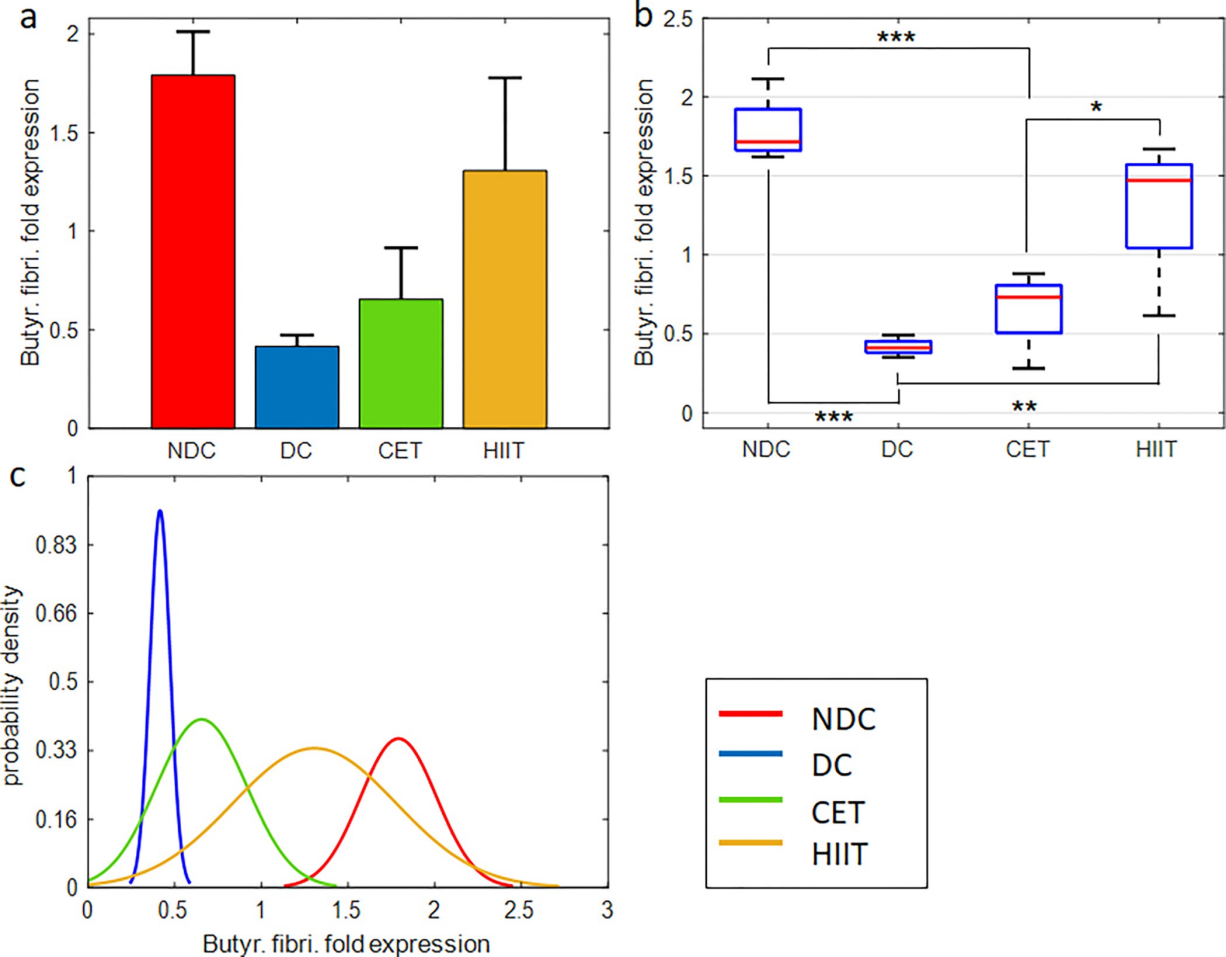
Exercise intervention effect on *Butyrivibrio fibrisolvens* expression in cecal tissue. (a,b) bar and box plots representing the relative value and variation span of *Butyrivibrio* expression in NDC, DC, CET, and HIIT groups. Medians in (b) are marked with red lines. Tukey’s multiple comparison test is used to determine the difference level between the exercise and control groups, ***p ≤ 0.001, **p ≤ 0.01, and *p ≤ 0.05, n = 8. The normalized relative gene expression values are obtained based on 2-ΔΔCT method, comparing target with universal bacterium. (c) Probability density function representing normal distribution of *Butyrivibrio* gene expression in the studied groups.

**Fig 6:**
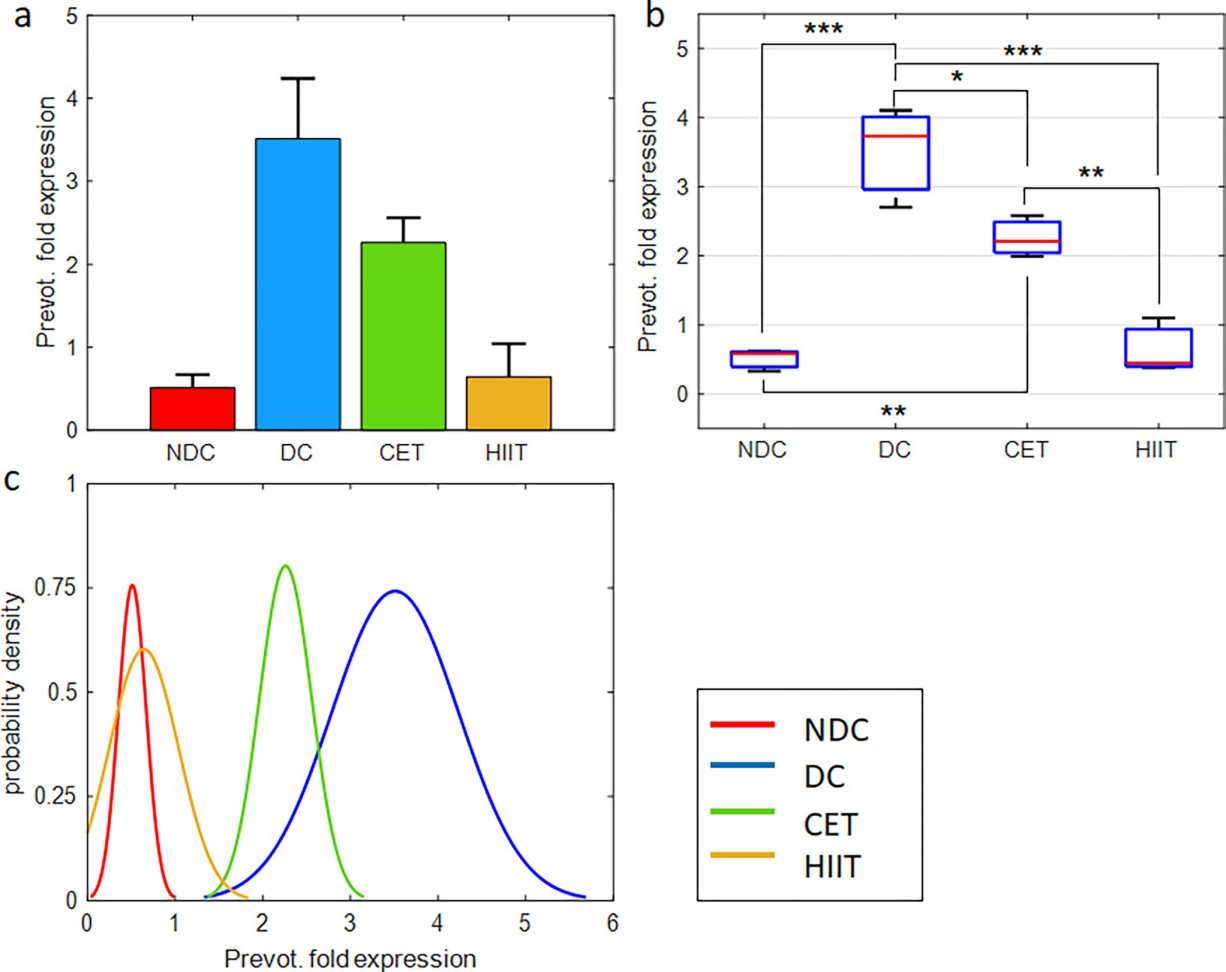
Exercise intervention effect on *Prevotella copri* expression in cecal tissue. (a,b) bar and box plots representing the relative value and variation span of *Prevotella* expression in NDC, DC, CET, and HIIT groups. Medians in (b) are marked with red lines. Tukey’s multiple comparison test is used to determine the difference level between the exercise and control groups, ***p ≤ 0.001, **p ≤ 0.01, and *p ≤ 0.05, n = 8. The normalized relative gene expression values are obtained based on 2-ΔΔCT method, comparing target with universal bacterium. (c) Probability density function representing normal distribution of *Prevotella* gene expression in the studied groups.

In this section, we describe the effect of diabetes and exercise on the expression of *Akkermansia muciniphila* and *Butyrivibrio fibrisolvens* microbes which are involved in the production of SCFAs such as butyrate [38, 47], and *Prevotella copri* as one of the main bacteria contributes in the production of BCAAs and the induction of insulin resistance [48] (Figs 4–6 and S9, S11 and S13 Tables).

#### Akkermansia muciniphila

As seen in Fig 4A, diabetes caused a 56% decrease in the expression of *Akkermansia* from 1.36 to 0.61. In the following, exercise intervention has compensated and increased the relative expression of this microbe up to 0.82 in CET exercise and 1.19 in HIIT exercise. In other words, CET and HIIT exercises have compensated for about 14.57, and 41.0% of the decrease in expression caused by diabetes, respectively. Therefore, it can be seen that the HIIT exercise pattern was successful in restoring the microbial population of *Akkermansia muciniphila* in the cecum tissue, while CET had no significant effect. The between-group comparison (Fig 4B and S9 Table) shows a significant difference in the performance of HIIT and CET exercises (P<0.01). Also, the restoration of *Akkermansia* gene expression in HIIT has been very successful and significant compared to the diabetic group. While the difference between the CET group and the diabetic group was not significant. Also, the one-way ANOVA confirms the hypothesis of the effectiveness of exercise intervention on the microbial abundance of *Akkermansia muciniphila* in the cecum (P = 6.96e-07, S10 Table). The probability density function (Fig 4C) shows the tendency of the microbial population of the CET group to be diabetic and the HIIT group to be non-diabetic. S9 Table shows the mean, STD, and SE by group.

#### Butyrivibrio fibrisolvens

Fig 5 and S11 Table show the effect of diabetes and exercise interventions on the relative expression of *Butyrivibrio fibrisolvens*. Considering that this microbe is one of the major producers of SCFAs in the intestine and plays a role in regulating glucose metabolism [39], it is crucial to know how diabetes and exercise interventions have affected its abundance. According to Fig 5A and 5B, diabetes caused a 77% decrease in the relative expression of *Butyrivibrio fibrisolvens* from 1.79 to 0.41. The negative effect of diabetes on the performance and abundance of this beneficial bacteria has also been reported in previous studies [39, 49]. Based on these observations, it can be expected that diabetes has a more destructive effect on the abundance of *Butyrivibrio* than *Akkermansia* (compare Figs 4A and 5A). Here, similar to the observations related to *Akkermansia*, exercise interventions have partially compensated for the negative effect of diabetes on the *Butyrivibrio* gene expression. To be more precise, the CET and HIIT groups have negated about 13.3% and 49.63% of the decrease in expression caused by diabetes, respectively. As can be seen, unlike CET, HIIT intervention was successful in restoring the microbial population of *Butyrivibrio* in the cecal tissue. Also, the comparison between CET and HIIT groups shows a significant difference (P = 0.036, Fig 5B and S11 Table). Based on one-way ANOVA, the hypothesis of the effectiveness of exercise interventions on the relative abundance of *Butyrivibrio fibrisolvens* is confirmed (P = 8.8e-5, S12 Table). The difference between gene expression of HIIT and non-diabetic groups was found to be non-significant, which indicates the successful performance of this exercise in recovering from the side effects of diabetes. This is while the difference between CET and NDC groups is high and at a significant level (P = 0.0006), which shows the weaker performance of this intervention in modulating the adverse effects of diabetes on *Butyrivibrio* bacteria. This issue is also evident in the probability density function (Fig 5C). The HIIT curve in this figure tends to NDC and CET to DC. Other statistical variables, including mean, STD, and SE with the removal of outlier data, are reported in S11 Table.

#### Prevotella copri

**Fig 7:**
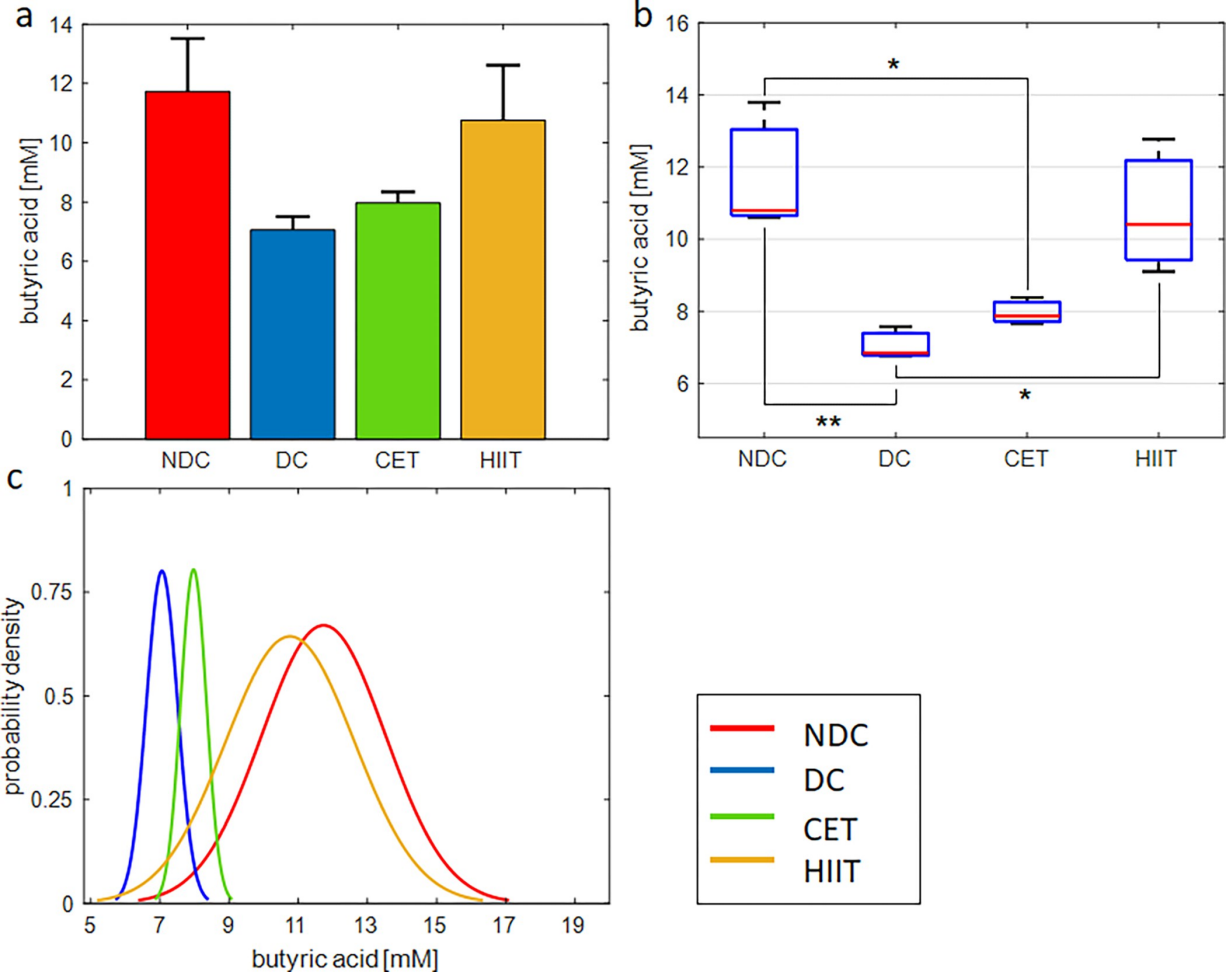
Exercise intervention effect on cecal concentration of butyrate. (a,b) bar and box plots representing the relative value and variation span of butyrate concentration in NDC, DC, CET, and HIIT groups. Medians in (b) are marked with red lines. Tukey’s multiple comparison test is used to determine the difference level between the exercise and control groups, ***p ≤ 0.001, **p ≤ 0.01, and *p ≤ 0.05, n = 8. (c) Probability density function representing normal distribution of butyrate concentration in the studied groups.

**Fig 8:**
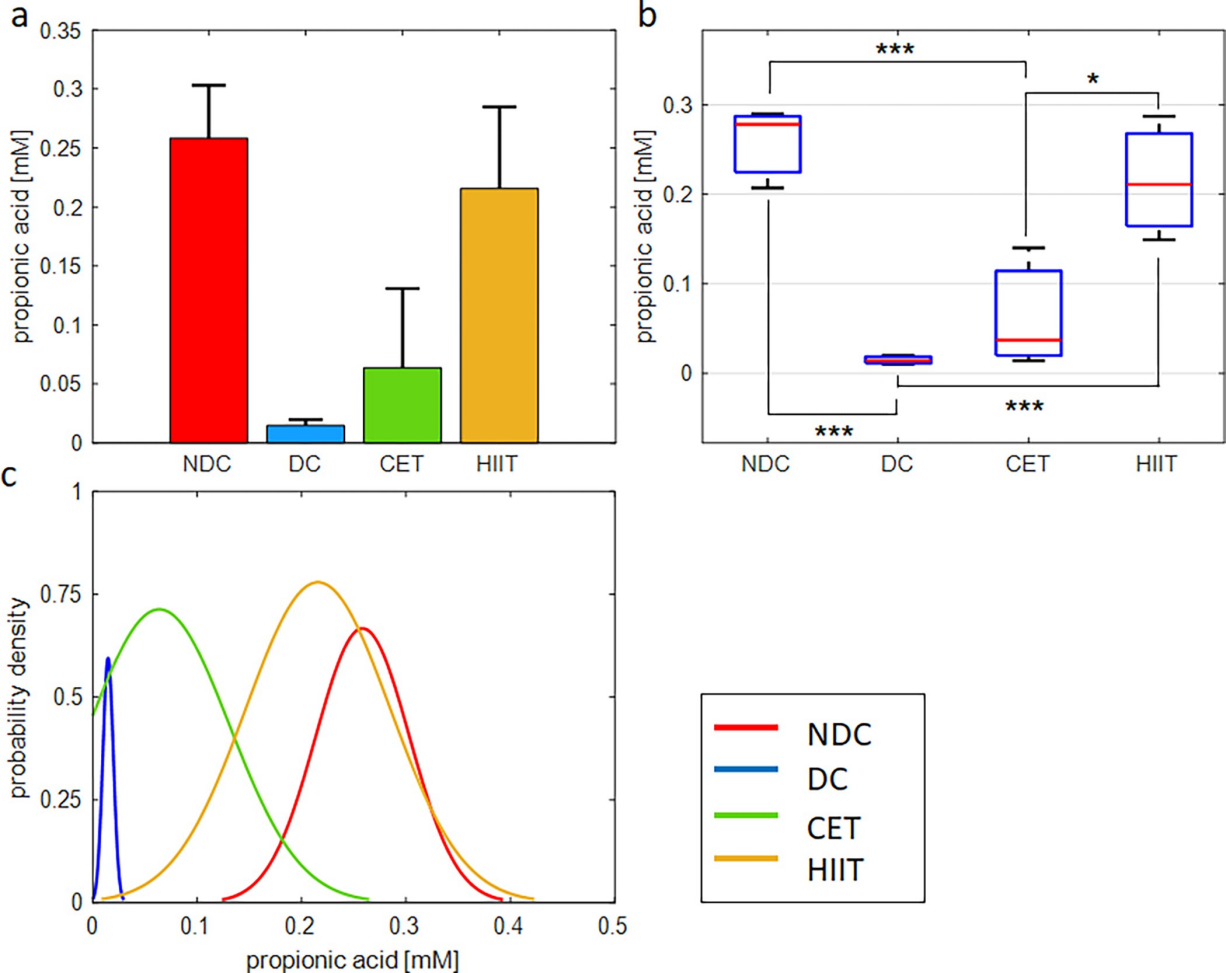
Exercise intervention effect on cecal concentration of propionate. (a,b) bar and box plots representing the relative value and variation span of propionate concentration in NDC, DC, CET, and HIIT groups. Medians in (b) are marked with red lines. Tukey’s multiple comparison test is used to determine the difference level between the exercise and control groups, ***p ≤ 0.001, **p ≤ 0.01, and *p ≤ 0.05, n = 8. (c) Probability density function representing normal distribution of propionate concentration in the studied groups.

The results of the qPCR test of *Prevotella copri* bacteria on the cecal tissue samples are shown in Fig 6 and S13 Table. The relative expression of *Prevotella* in the diabetic group has increased seven times from 0.51 to 3.51. Considering the counteracting role of this bacterium in the production of SCFAs reported in previous studies [50], it can be expected that its multifold increase in DC group will cause dysbiosis of the gut microbiome and disrupt the production of diabetes-modulating metabolites (Note the bacterial and metabolic perturbations of DC group in other sections as well (Figs 4, 5, 7 and 8)). In the following, the application of exercise interventions has caused a decrease in *Prevotella* expression (Fig 6A) to the extent that the HIIT group reached very close to the non-diabetic normal condition. The comparison between CET and HIIT groups shows a significant difference in the effectiveness of HIIT in modulating the expression of *Prevotella copri* gene (Fig 6B and S13 Table). Here, as in the previous sections, the CET pattern has less effect on the intestinal microbial content. Based on the results obtained from one-way ANOVA, the hypothesis of the effectiveness of exercise interventions and diabetes on *Prevotella copri* microbe is confirmed (P = 9.76e-5, S14 Table). The probability density function of HIIT is very close and converged to the non-diabetic group, however, the probability density function of the CET group is between diabetic and non-diabetic and tends to the diabetic group. Quantitative statistics of average, STD, and SE by groups are listed in S13 Table.

### The effect of exercise on microbial metabolites (SCFAs)

Among SCFAs effective in metabolic syndrome, butyric and propionic acids are selected and measured by the HPLC method. Considering that *Akkermansia* and *Butyrivibrio* are known as two of the bacteria that produce the above metabolites [47, 51], the results of this section can be used indirectly in the cross-validation of the previous part, "Effect of exercise on intestinal microbial content". In addition, these results can help us find the impact of diabetes and exercise interventions on the concentration of SCFAs in the intestinal tissue.

#### Butyric acid

The results of measuring cecal butyric acid concentration are shown in Fig 7 and S15 Table. Based on Fig 7A, the concentration of butyric acid in the diabetic group has decreased from 11.73 mM to 7.05 mM. This result is consistent with previous observations regarding the negative effect of diabetes on SCFAs [3]. In addition, HIIT intervention has improved the concentration of butyric acid significantly. Nevertheless, CET exercise had no significant effect on butyrate levels. The multiple comparisons test shows a significant difference between diabetic groups with HIIT (P = 0.036) and non-diabetic groups with CET (P = 0.033). Also, based on the one-way ANOVA test, the effectiveness of exercise interventions on butyric acid concentration is confirmed (P = 0.0073, S16 Table). Based on Fig 7C, the probability density function of the CET group is biased towards DC and HIIT towards NDC. The STD of the non-diabetic and HIIT groups is around 1.8 mM, and the STD of the diabetic and CET groups is about 0.4 (S15 Table).

#### Propionic acid

Changes in propionic acid concentration caused by diabetes and exercise are shown in Fig 8 and S17 Table. As can be seen, propionate concentration has dropped heavily under the influence of diabetes (94% compared to the non-diabetic group). With exercise intervention, the concentration of this fatty acid has increased (CET, 0.6 mM and HIIT, 0.21 mM). Similar to the butyrate results, the HIIT exercise pattern has shown better performance in improving propionate concentration than CET. The performance difference between CET and HIIT was obtained at a significant level of 0.032. The one-way ANOVA hypothesis test confirms the effect of diabetes and exercise interventions on the cecal concentration of propionic acid (P = 0.0013, S18 Table). The probability density function of the HIIT group overlaps with non-diabetic subjects and the CET group with diabetics. Statistical measures of mean, STD, and SE by groups are shown in S17 Table.

## Discussion

In this study, the effect of two types of exercises: high-intensity interval and continuous endurance on the manifestations of metabolic syndromes, such as type 2 diabetes and inflammatory factors, was investigated. For this purpose, inflammatory factors IL6, TLR4, and intestinal microbial content and metabolites resulting from their activity were measured in diabetic rats induced by a high-fat diet. The results showed that exercise effectively reduces the inflammatory factors and regulates the intestinal microbial population. Among the investigated microbes, the relative expression of two microbes, *Akkermansia muciniphila* and *Butyrivibrio fibrisolvens*, which represent the production of SCFAs, increased. On the other hand, the relative abundance of *Prevotella Copri* bacteria, representative of BCAA production, which induces insulin resistance, has decreased. In line with the above observations, an increase in butyrate and propionate metabolites caused by the fermentation of the microbial populations of *Akkermansia muciniphila* and *Butyrivibrio fibrisolvens* was observed in the cecal tissue. Various measurement methods were chosen for tissue and serum samples with the aim of cross-validation the results so that the results of all three sections of the microbial, metabolic, and inflammatory analysis confirm each other. Among the two exercise patterns, CET has a more significant influence on reducing IL6 and TLR4 inflammatory factors (see the p-values in S5 and S7 Tables). Therefore, it can be expected that CET exercise is a more successful model in controlling inflammation as one of the factors involved in metabolic syndrome diseases. Of course, any definite conclusion about this requires the analysis of more cytokines. At the same time, HIIT exercise has been more successful in increasing the population of bacteria representing SCFAs, modulating fasting blood sugar and insulin resistance. This promising performance may be due to the anaerobic nature of this sports model because the studied microbes are also anaerobic [52]. However, the correlation between the anaerobicity of the exercise pattern and the microbial population requires more extensive research. The difference in the effect of CET and HIIT exercise patterns on modulating inflammatory, microbial, and metabolic factors makes it impossible to suggest one of these two as an absolute solution to control all the symptoms of metabolic syndrome. In this study, the differential effects of two exercise patterns were investigated. Yet, in future supplementary studies, the combined effects of these two patterns and even other exercise patterns can be investigated.

In line with the present study, other studies also looked at the effect of a high-fat diet on inflammatory pathways. For example, high-fat food induces inflammation through increasing endotoxin in the intestinal lumen and blood plasma [12, 53]. On the other hand, increasing the level of TLR4 has led to an increase in intestinal permeability and acceleration of obesity [54]. The modulating effect of exercise on intestinal microbial profile has also been shown to weaken inflammatory pathways in obese children [55]. In this study, implementing 12 weeks of a combined endurance-resistance program has led to a significant decrease in *Proteobacteria* and an increase in *Blautia*, *Dialister*, and *Roseburia* bacteria (from the *Firmicute* family). These results are consistent with the changes recorded in our research on two bacteria, *Akkermansia muciniphila* and *Butyrivibrio fibrisolvens*. Also, studies have shown that the voluntary running of rats leads to an increase in butyrate concentration and cecum diameter [56–58]. These studies stated that the amount of physical activity performed has an inverse relationship with the ratio of *Bacteroides* to *Firmicutes*. This inverse relationship was also seen in our research, as doing sports activities increased the expression of *Akkermansia muciniphila* and *Butyrivibrio fibrisolvens* (representing the *Firmicute* family) and decreased *Prevotella Copri* representing the *Bacteroides*. On the other hand, the improvement of the intestinal microbial profile to a healthy state due to exercise has been observed in our research and some previous studies [57, 59, 60]. The effect of exercise on some inflammatory parameters has been studied so far. For example, performing 8 weeks of HIIT has led to a significant decrease in the serum concentration of tumor necrosis factor-α (TNF-α) and chemerin, and improved body composition and insulin resistance in obese women [61]. Also, regular HIIT has been shown to reduce TNF-α levels and slow down the progression of Parkinson’s disease by improving serum antioxidant capacity [62]. In another study, endurance exercise in mice fed a high-fat diet (HFD) caused a significant decrease in the serum levels of fatty acids, ceramides, expression of TNF-α, IL-18, and macrophage inflammatory protein-1γ (MIP-1γ) [63]. These observations suggest that endurance exercise may help to improve HFD-induced complications by reducing ceramides, reducing inflammasome activation in adipose tissue, and systemic downregulating inflammatory cytokines. Moreover, the positive effect of different endurance and resistance training patterns on the modulation of other pro-inflammatory cytokines such as IL-1β and adipocytokines such as leptin, adiponectin, and resistin has been reported [64–66].

In metagenomic studies in animal models, mainly lab mice, rats, and chickens, it has been found that while some bacterial species such as *Akkermansia* and *Proteobacteria*, *Faecalibacterium prausnitzii*, *Acinetobacter*, *Fusobacterium*, *Bifidobacterium* respond to exercise, others are less affected [22]. Likewise, the three bacterial species in our study did not respond equally to exercise. Recent research has also shown that exercise can stimulate genes encoding the production of SCFAs such as acetate, butyrate, and propionate [59, 67, 68]. The results of measuring the concentration of two important metabolites of SCFAs, including propionate and butyrate, in the present study confirmed this issue.

### Limitations

Notably, the use of animal models, despite their advantages in the controllability of testing processes, faces limitations in generalizing the results to humans, for which it is necessary to conduct additional clinical trials. Variation of exercise parameters was another challenge. Specifically, choosing the duration, number, and intervals of exercise sessions, speed and pressure of movements, and even the possibility of combining several patterns can be considered. Faced with diverse exercise patterns, we used the two main and well-known categories of HIIT and CET as representatives of anaerobic and endurance movements with the standard protocol mentioned in the references [52, 69, 70]. Using other known exercise patterns or their combination in future studies can help further clarify the effect of exercise on metabolic syndrome. The presence of numerous and diverse microbial species in the intestine made it hard to choose the appropriate strain for this research. Among the different species, three important bacteria named *Akkermansia muciniphila*, *Butyrivibrio fibrisolvens*, and *Prevotella Copri*, which represent the *Firmicute* and *Bacteroid* families and are involved in the production of SCFAs and BCAAs, were selected. Of course, achieving more comprehensive results requires the investigation of other bacterial species involved in metabolic syndrome. It is noteworthy that the type of sample used may also affect the test result. Hence, in this study, a collection of serum and cecum data was analyzed. Additional studies can be done on liver, kidney, feces, and other organs involved in metabolic syndrome. Finally, in the future, investigating other inflammatory factors in addition to TLR4 and IL6, e.g., TNF-α, IL-1β, Interferon-γ (IFN‐γ), and nuclear factor kappa B (NF-κB), can lead to a more comprehensive understanding of the relationship between exercise and inflammation.

## Concluding remarks

In this study, we compared the effect of continuous endurance and high-intensity interval exercises on cecal microbial metabolites and sera inflammatory factors in diabetic Wistar rats. The results showed that HIIT outperforms CET in regulating fasting blood sugar, balancing Firmicute to Bacteroid ratio, and increasing SCFAs metabolites. In particular, HIIT caused a significant increase in the expression of *Akkermansia* and *Butyrivibrio* bacteria and their metabolites, including butyrate and propionate, and modulating the expression of *Prevotella* bacteria. These observations can indicate the effectiveness of HIIT on improving the digestive system and reducing insulin resistance [16, 17, 48]. On the other hand, CET has been more successful in reducing Il6 and TLR4 inflammatory factors. These biomarkers are located in the upstream of the inflammation cascade and the activity of many other inflammatory factors directly or indirectly depends on them [27, 28]. In addition, these two factors play an important role in regulating the rate of intestinal permeability and controlling obesity [29–31]. Despite this key role, any final conclusion about the effect of the studied exercises on inflammation requires testing of other cytokines.

The results of this research, while emphasizing the importance of the relationship between exercise and gut microbiome, can be used to provide a suitable exercise protocol among two endurance and intensity groups to prevent disorders induced by a high-fat diet. Also, this research helps to show a clearer picture of the therapeutic effect of exercise on modulating the intestinal microbial population, eliminating dysbiosis, and controlling type 2 diabetes.

## Supporting information

S1 Checklist *PLOS ONE* humane endpoints checklist. (PDF)

S1 Table Sequence of primers used to investigate TLR4 gene expression. (TIF)

S2 Table 16s rRNA primers of the studied bacteria. (TIF)

S3 Table Calibrators preparation. (TIF)

S4 Table Mobile phase transition program by gradient method. (TIF)

S5 Table Intergroup comparison test of IL6 along with the statistical characteristics of each group. (TIF)

S6 Table Investigating the hypothesis of the effect of exercise interventions on sera IL6 concentration using one-way ANOVA. (TIF)

S7 Table Intergroup comparison test of TLR4 along with the statistical characteristics of each group. (TIF)

S8 Table Investigating the hypothesis of the effect of exercise interventions on cecal TLR4 expression using one-way ANOVA. (TIF)

S9 Table Intergroup comparison test of *Akkermansia* along with the statistical characteristics of each group. (TIF)

S10 Table Investigating the hypothesis of the effect of exercise interventions on cecal *Akkermansia* expression using one-way ANOVA. (TIF)

S11 Table Intergroup comparison test of *Butyrivibrio* along with the statistical characteristics of each group. (TIF)

S12 Table Investigating the hypothesis of the effect of exercise interventions on cecal *Butyrivibrio* expression using one-way ANOVA. (TIF)

S13 Table Intergroup comparison test of *Prevotella* along with the statistical characteristics of each group. (TIF)

S14 Table Investigating the hypothesis of the effect of exercise interventions on cecal *Prevotella* expression using one-way ANOVA. (TIF)

S15 Table Intergroup comparison test of butyrate along with the statistical characteristics of each group. (TIF)

S16 Table Investigating the hypothesis of the effect of exercise interventions on cecal butyrate concentration using one-way ANOVA. (TIF)

S17 Table Intergroup comparison test of propionate along with the statistical characteristics of each group. (TIF)

S18 Table Investigating the hypothesis of the effect of exercise interventions on cecal propionate concentration using one-way ANOVA. (TIF)

S1 File (DOCX)
