# Supplementary material for: Efficacy of high-intensity interval and continuous endurance trainings on cecal microbiota metabolites and inflammatory factors in diabetic rats induced by high-fat diet
Source: PLoS One. 2024 Apr 16;19(4):e0301532. doi: 10.1371/journal.pone.0301532 (PMC11020751; doi:10.1371/journal.pone.0301532)
Supplement: S1 Checklist — (PDF) [file pone.0301532.s001.pdf]

PLOS ONE Humane Endpoints Checklist

PLOS ONE manuscript number: PONE-D-23-16405

Complete the following if your study design includes death of a regulated animal as a likely outcome or planned experimental endpoint. Please also include all information in the Methods section of your manuscript.

ITEM 1. Describe whether humane endpoints* were used for all animals involved in the study.

	Recommendation	Section/Paragraph
If humane endpoints were used, report the following:*		
1	The specific criteria used to determine when animals should be euthanized	48 hours after the last exercise session
2	Once animals reached endpoint criteria, the amount of time elapsed before euthanasia	6 hours
3	Whether any animals died before meeting criteria for euthanasia	Yes, 5 of the diabetic rats died due to leg injuries and accidents during exercise.
If humane endpoints were **not** used, report the following:*		
1	A scientific and ethical justification for the study design, including the reasons why humane endpoints could not be used, and discussion of alternatives that were considered but could not be used	---
2	Whether the institutional animal ethics committee specifically reviewed and approved the anticipated mortality in the study design	---

ITEM 2. Include the following details of the study design and outcomes.

	Recommendation	Section/Paragraph
1	The duration of the experiment	one week of acclimatization to the environment<br>+<br>a 16-week high-calorie diet to induce diabetes<br>+<br>One week to familiarize with the exercise protocol after the induction of diabetes<br>+

		Nine weeks implementation of the exercise protocol<br>-----<br>Total clinical experiment duration: 27 weeks
2	The numbers of animals used, euthanized, and found dead (if any); the cause of death for all animals	❖ Number of used animals: 45 male Wistar rats<br>❖ Number of animals excluded from the experiment process: 13 rats:<br>1) 8 rats were excluded due to not reaching the diabetes threshold<br>2) 5 diabetic rats were anesthetized and euthanized immediately after leg injury during exercise so as not to suffer from diabetic wound pain.<br>❖ Number of euthanized animals: 37 rats
3	How frequently animal health and behavior were monitored	The general condition and appearance of the rats were checked daily. The health status of the rats, their activity and feeding have been checked daily. Fasting blood sugar, weight and body mass index were checked at the beginning and end of the experiment, as well as before and after the induction of diabetes and after performing the exercise protocol.
4	All animal welfare considerations taken, including efforts to minimize suffering and distress, use of analgesics or anaesthetics, or special housing conditions	Animals were housed individually in cages in a room that was controlled for temperature (22 °C), humidity and light (12 h light: dark cycle). The animal housing was well ventilated.<br>All rats had free access to laboratory chow and tap water ad libitum. Food and water containers were sterilized daily. The health status, activity and eating of the rats were monitored daily. The animal food was dry and high in fat, and to prevent it from spoiling, it was kept in a freezer at -18 degrees, and the rest was kept in the refrigerator while it was being used. The water, food and bedding of the animals have been changed every other day. All animal treatments were considered humanly and in compliance with the recommendation of the animal care committee of AJA University of Medical Sciences and the principles of laboratory animal care (Approval Code: IR.AJA UMS REC 1400.287).

		Before starting the experiment, the rats were given enough time to familiarize themselves with the environment and training conditions. After 24 h of the last training session and after an overnight fasting, the rats were taken one by one to the dissection room and anesthetized by intra peritoneal injection of ketamine (90 mg per kg of body weight) and xylazine (10 mg per kg of body weight). All procedures were carried out under the supervision of laboratory animal care experts. After anesthetization, the blood is collected directly and completely from the heart of the rats, which leads to the death of the animal. Next, the animal is dissected and the tissue of the cecum is kept in liquid nitrogen for further study. The serum sample is stored at -80 degrees. Disposable surgical blade and autoclaved surgical set have been used for dissecting. The rest of the animal carcasses were collected in special infectious garbage bags and bins and delivered to the incinerator department for disposal.
5	Any special training in animal care or handling provided for research staff	All the experimental steps were carried out under the supervision of animal care experts and the ethics committee of AJA University of Medical Sciences and based on the latest national guidelines for animal care[1].

Reference:
Ahmadi-Noorbakhsh, S., Mirabzadeh Ardakani, E., Sadighi, J. et al. **Guideline for the Care and Use of Laboratory Animals in Iran**. Lab Anim 50, 303–305 (2021). https://doi.org/10.1038/s41684-021-00871-3

***Definition of a humane endpoint**

A humane endpoint is an experimental endpoint at which animals are euthanized when they display early markers associated with death or poor prognosis of quality of life, or specific signs of severe suffering or distress. Humane endpoints are used as an alternative to allowing such conditions to continue or progress to death following the experimental intervention ("death as an endpoint"), or only euthanizing animals at the end of an experiment. Before a study begins, researchers define the practical observations or measurements that will be used during the study to recognize a humane endpoint, based on anticipated clinical, physiological, and behavioral signs. These may include, for instance, body temperature or weight changes, tumor size or appearance, abnormal behaviors, pathological changes, ruffled fur, reduced mobility, body posture, or expression of specific body fluid markers. Please see the NC3Rs guidelines for more information.

ARRIVE Guidelines

PLOS ONE encourages authors to follow the Animal Research: Reporting of In Vivo Experiments (ARRIVE) guidelines for all submissions describing laboratory-based animal research and to upload a completed ARRIVE Guidelines Checklist to be published as supporting information.
