# Supplementary figures and images for: Efficacy of high-intensity interval and continuous endurance trainings on cecal microbiota metabolites and inflammatory factors in diabetic rats induced by high-fat diet
Source: PLoS One. 2024 Apr 16;19(4):e0301532. doi: 10.1371/journal.pone.0301532 (PMC11020751; doi:10.1371/journal.pone.0301532)

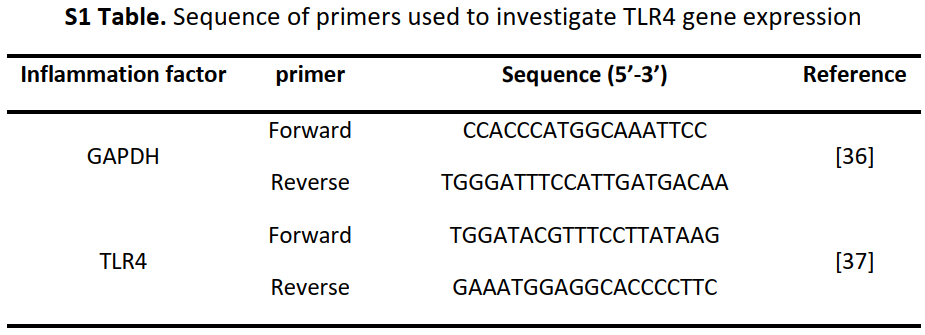

Supplement: S1 Table — (TIF) [file pone.0301532.s002.tif]

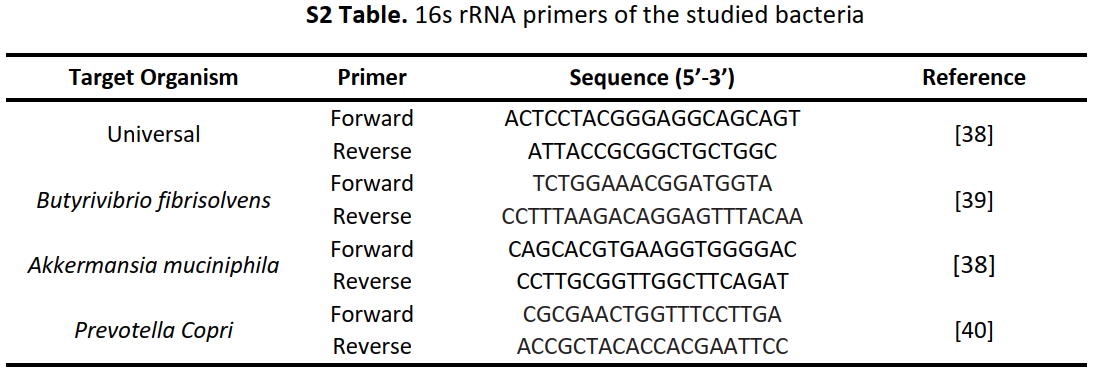

Supplement: S2 Table — (TIF) [file pone.0301532.s003.tif]

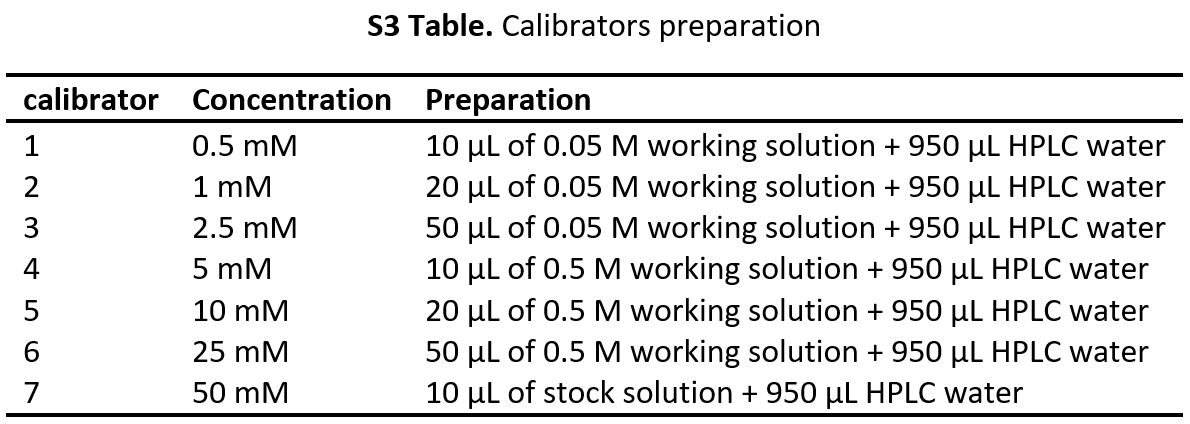

Supplement: S3 Table — (TIF) [file pone.0301532.s004.tif]

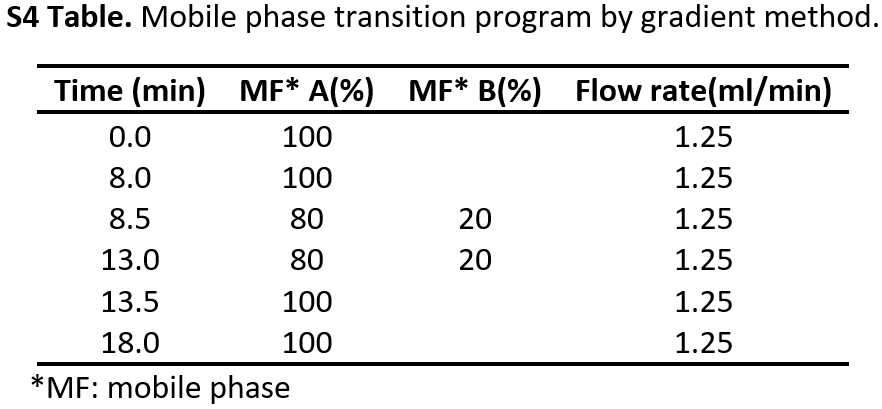

Supplement: S4 Table — (TIF) [file pone.0301532.s005.tif]

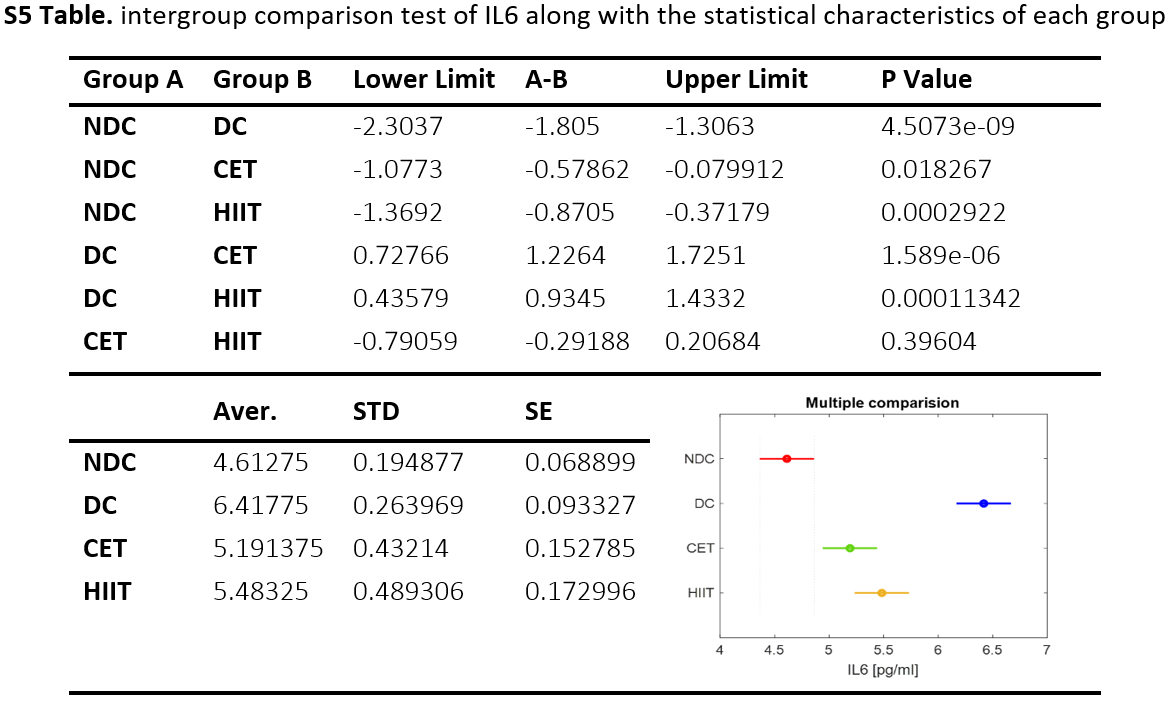

Supplement: S5 Table — (TIF) [file pone.0301532.s006.tif]

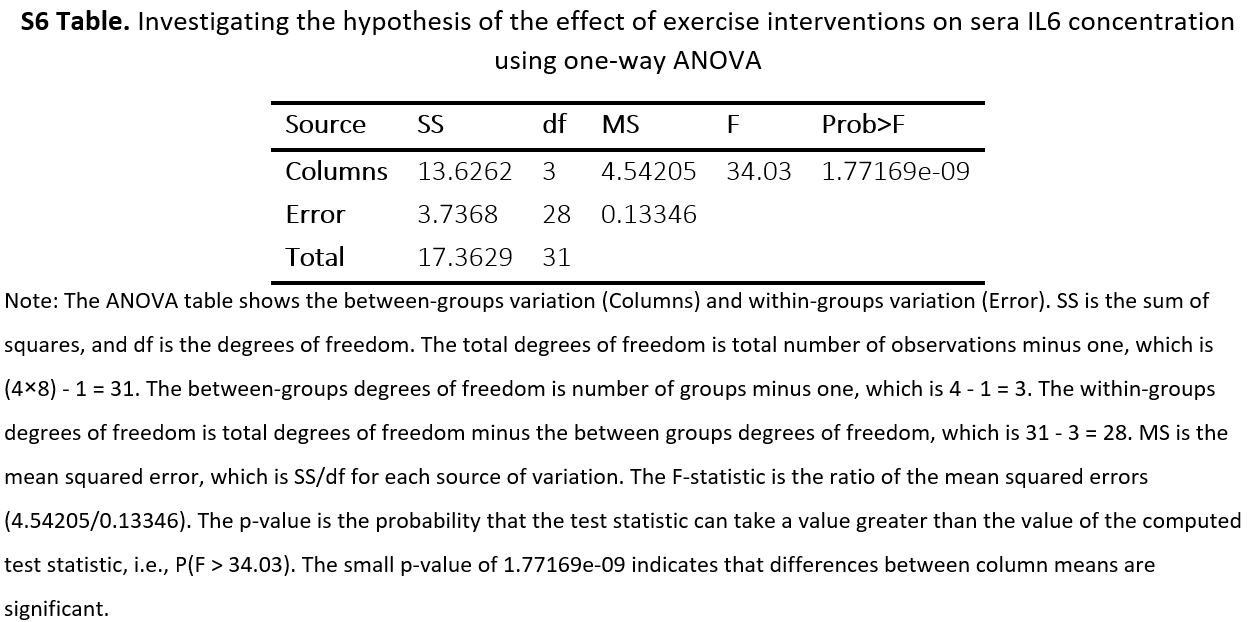

Supplement: S6 Table — (TIF) [file pone.0301532.s007.tif]

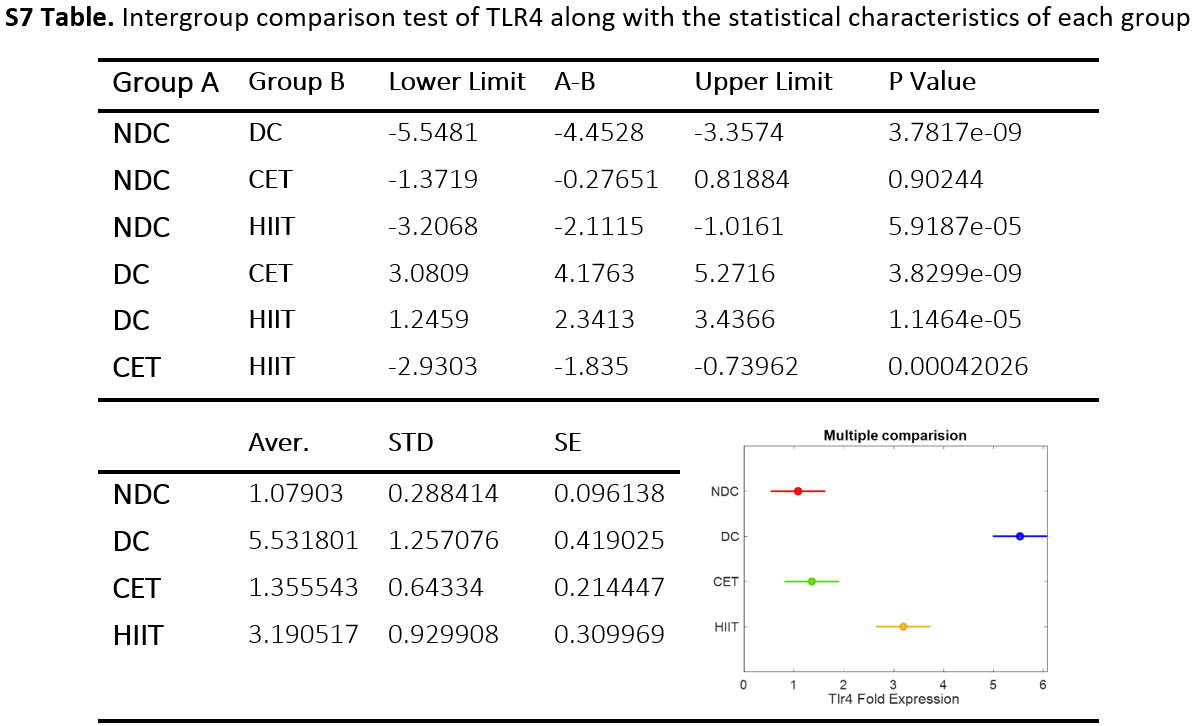

Supplement: S7 Table — (TIF) [file pone.0301532.s008.tif]

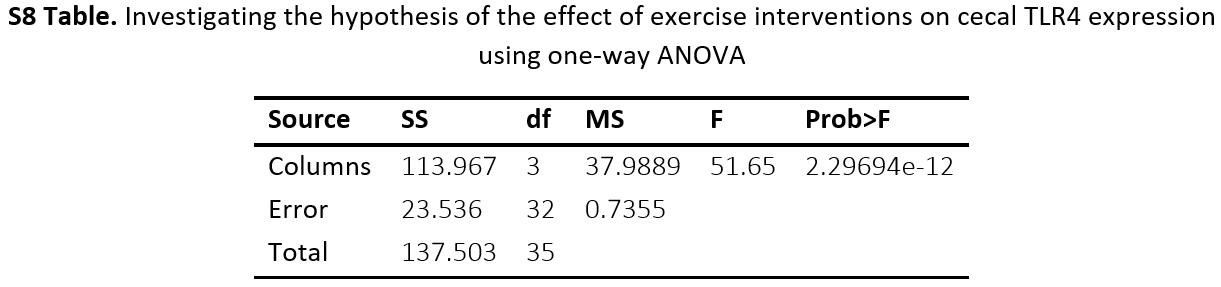

Supplement: S8 Table — (TIF) [file pone.0301532.s009.tif]

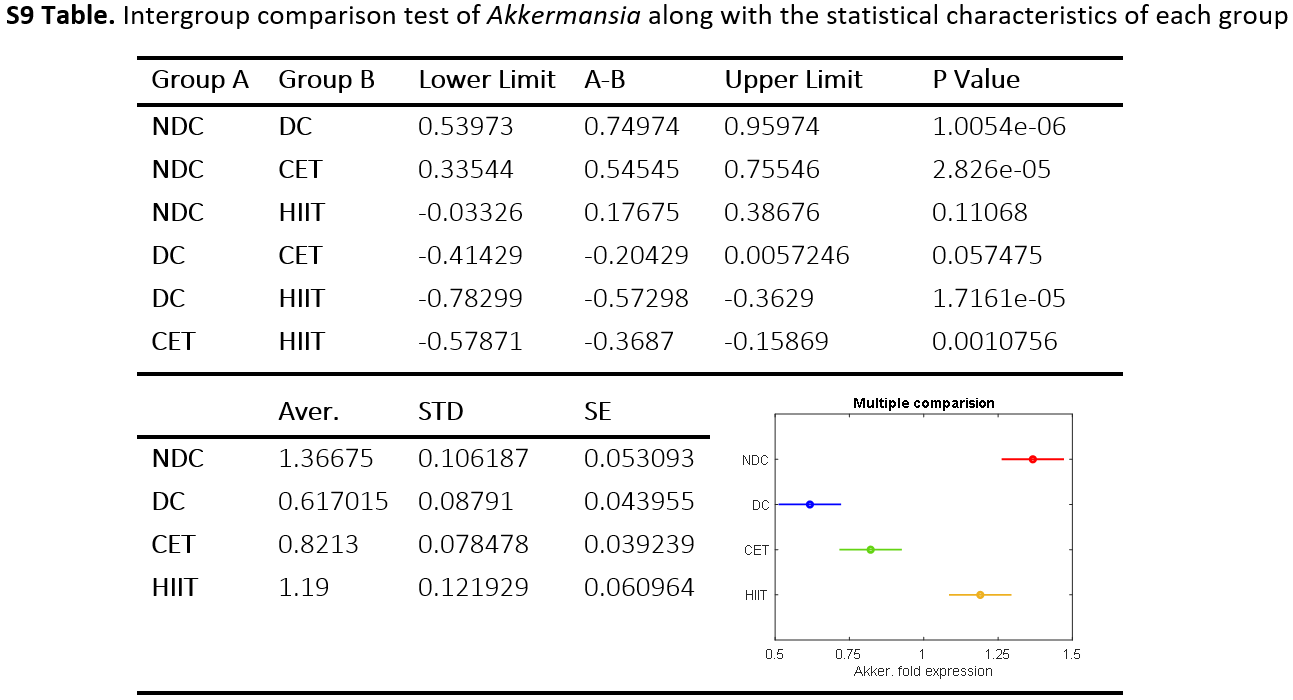

Supplement: S9 Table — (TIF) [file pone.0301532.s010.tif]

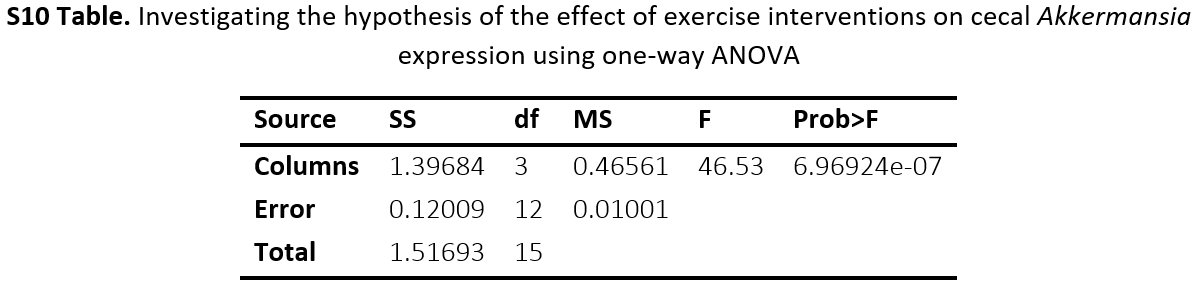

Supplement: S10 Table — (TIF) [file pone.0301532.s011.tif]

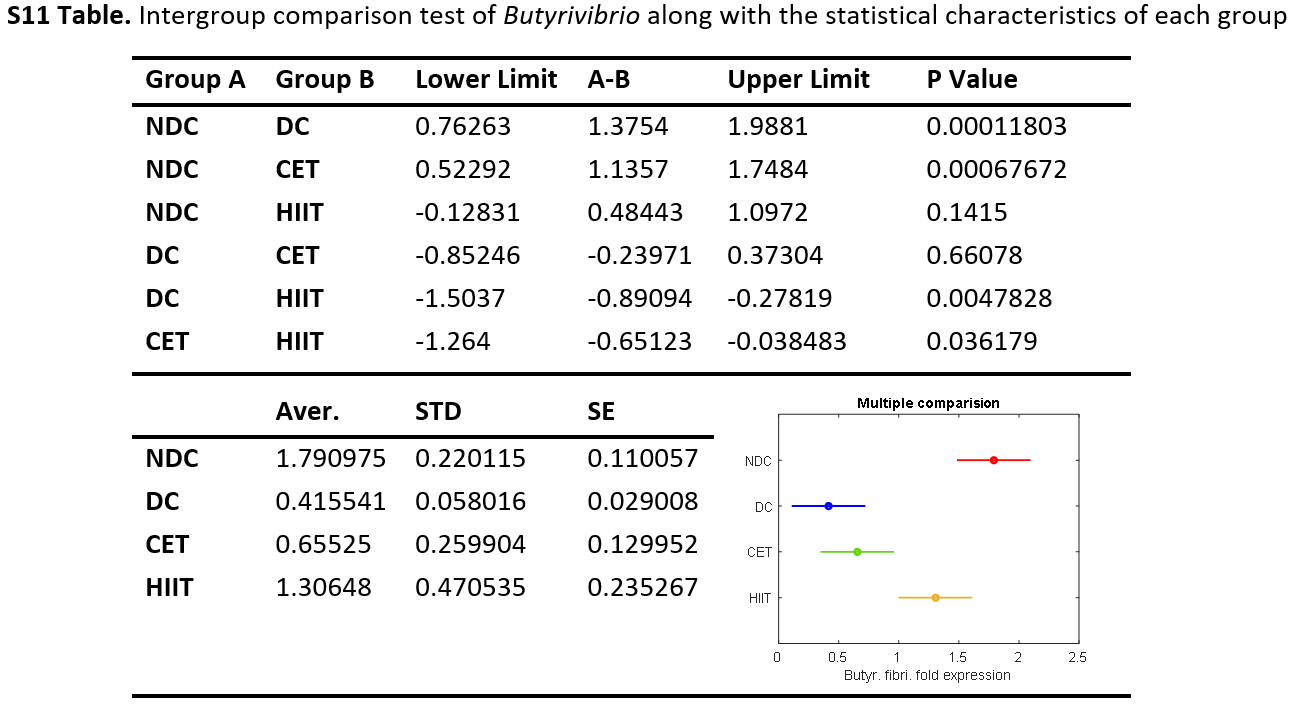

Supplement: S11 Table — (TIF) [file pone.0301532.s012.tif]

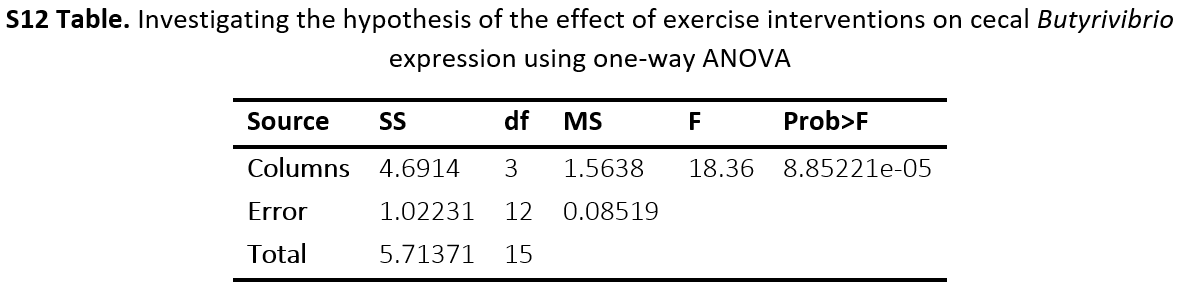

Supplement: S12 Table — (TIF) [file pone.0301532.s013.tif]

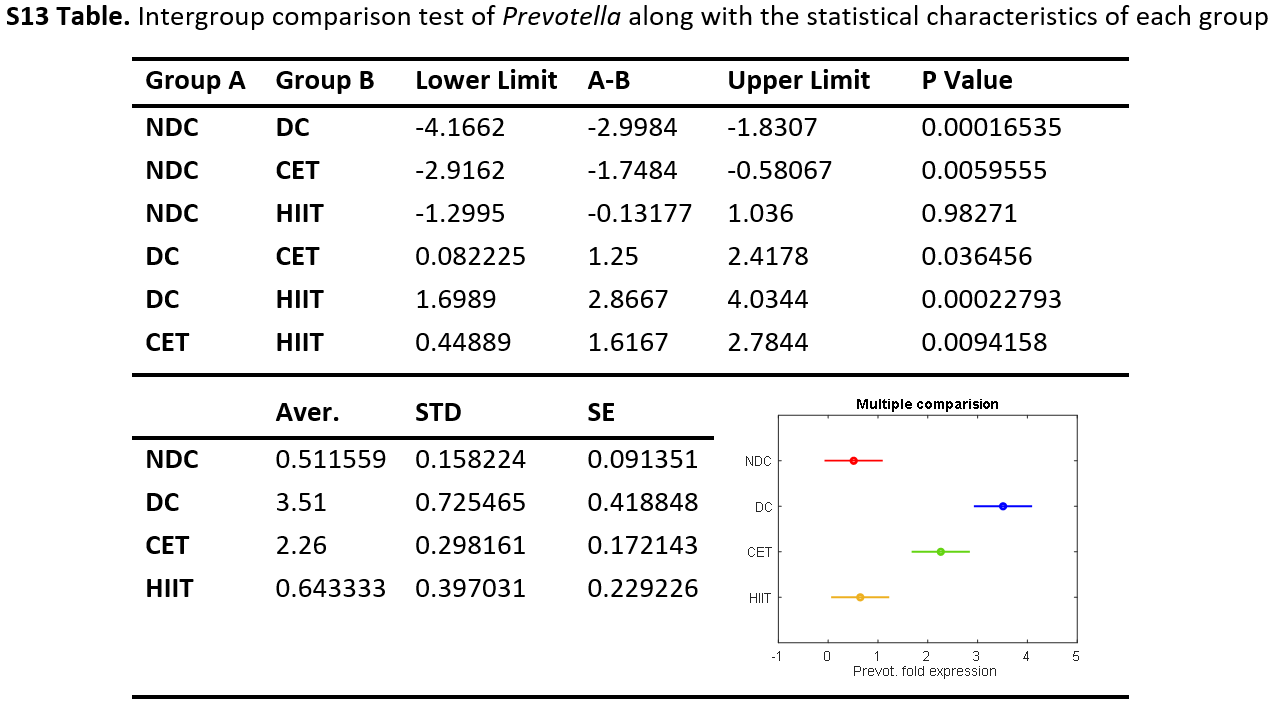

Supplement: S13 Table — (TIF) [file pone.0301532.s014.tif]

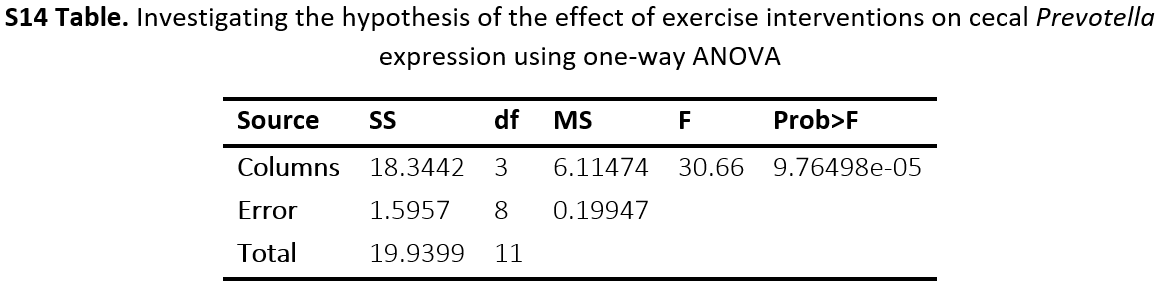

Supplement: S14 Table — (TIF) [file pone.0301532.s015.tif]

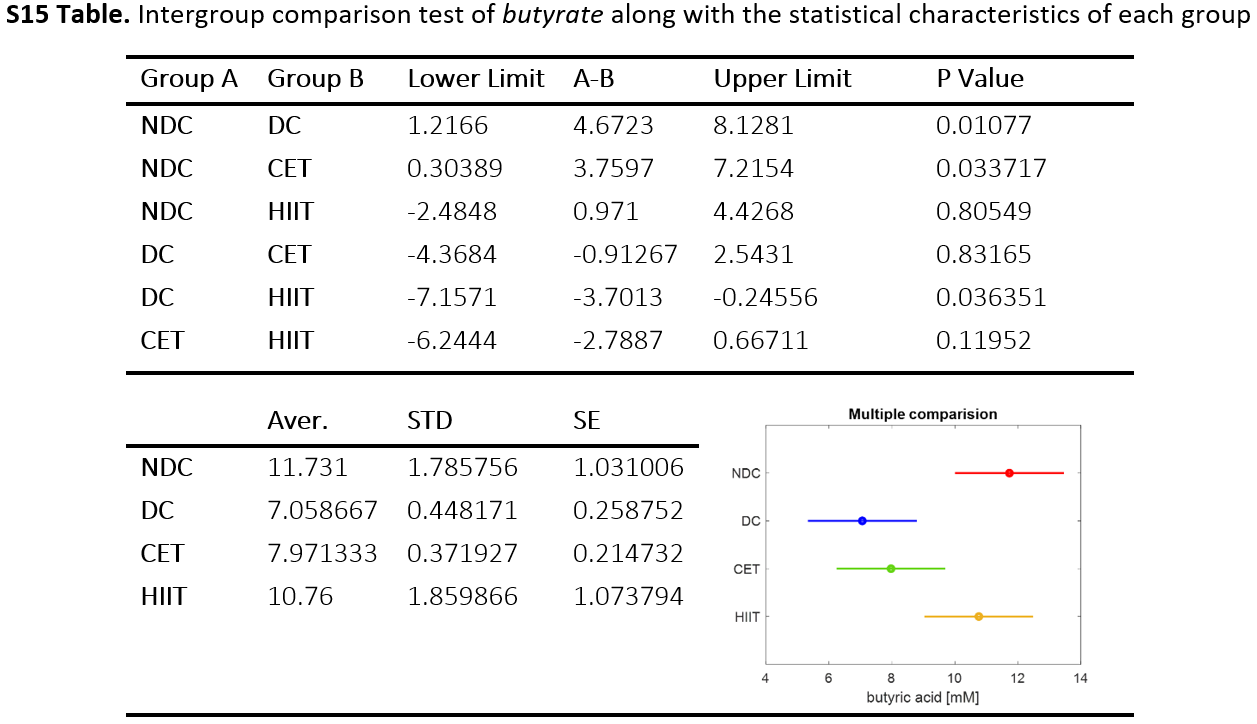

Supplement: S15 Table — (TIF) [file pone.0301532.s016.tif]

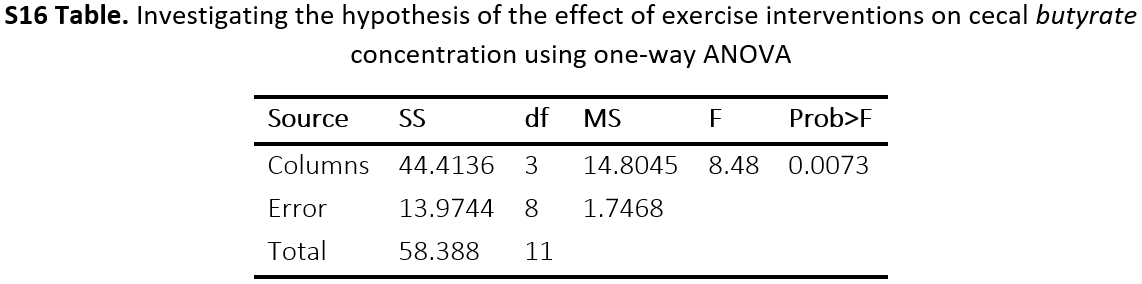

Supplement: S16 Table — (TIF) [file pone.0301532.s017.tif]

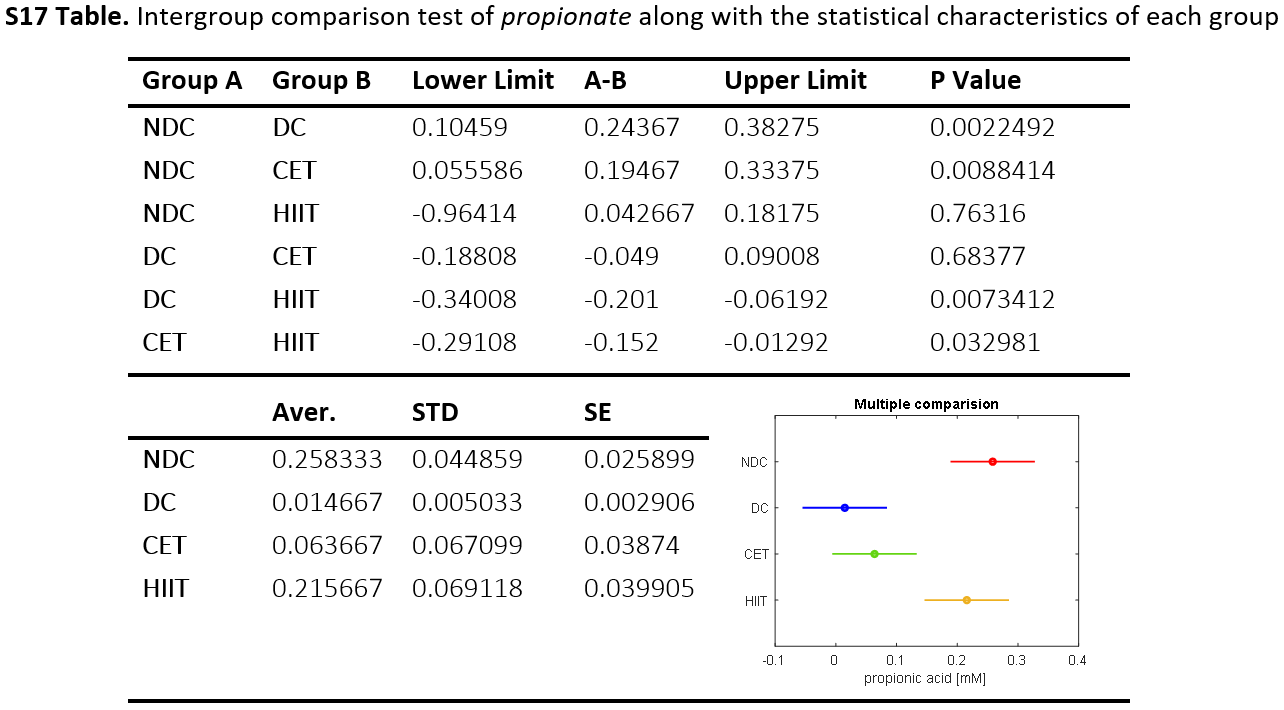

Supplement: S17 Table — (TIF) [file pone.0301532.s018.tif]

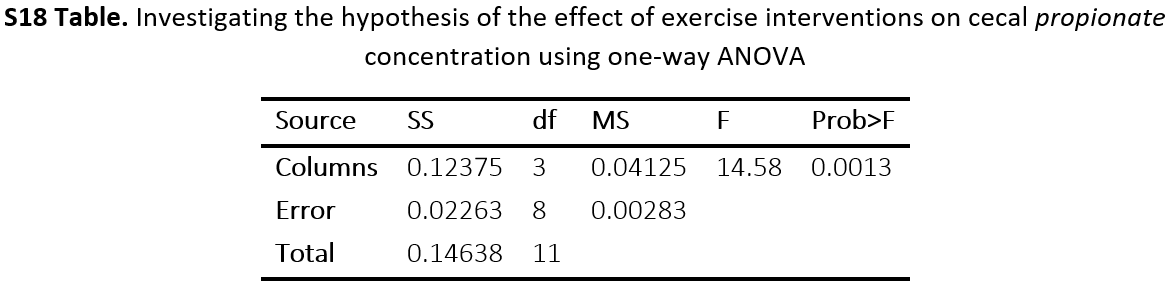

Supplement: S18 Table — (TIF) [file pone.0301532.s019.tif]
