# Supplementary material for: Efficacy of high-intensity interval and continuous endurance trainings on cecal microbiota metabolites and inflammatory factors in diabetic rats induced by high-fat diet
Source: PLoS One. 2024 Apr 16;19(4):e0301532. doi: 10.1371/journal.pone.0301532 (PMC11020751; doi:10.1371/journal.pone.0301532)
Supplement: S1 File — (DOCX) [file pone.0301532.s020.docx]

**Primers' sequence**

**S1 Table.** Sequence of primers used to investigate TLR4 gene expression

| **Reference** | **Sequence (5’-3’)** | **primer** | **Inflammation factor** |
| --- | --- | --- | --- |
| [36] | CCACCCATGGCAAATTCC  TGGGATTTCCATTGATGACAA | Forward  Reverse | GAPDH |
| [37] | TGGATACGTTTCCTTATAAG  GAAATGGAGGCACCCCTTC | Forward  Reverse | TLR4 |

**S2 Table.** 16s rRNA primers of the studied bacteria

| **Reference** | **Sequence (5’-3’)** | **Primer** | **Target Organism** |
| --- | --- | --- | --- |
| [38] | ACTCCTACGGGAGGCAGCAGT  ATTACCGCGGCTGCTGGC | Forward  Reverse | Universal |
| [39] | TCTGGAAACGGATGGTA  CCTTTAAGACAGGAGTTTACAA | Forward  Reverse | *Butyrivibrio fibrisolvens* |
| [38] | CAGCACGTGAAGGTGGGGAC  CCTTGCGGTTGGCTTCAGAT | Forward  Reverse | *Akkermansia muciniphila* |
| [40] | CGCGAACTGGTTTCCTTGA  ACCGCTACACCACGAATTCC | Forward  Reverse | *Prevotella Copri* |

**HPLC details**

**S3 Table.** Calibrators preparation

| **calibrator** | **Concentration** | **Preparation** |
| --- | --- | --- |
| 1 | 0.5 mM | 10 µL of 0.05 M working solution + 950 µL HPLC water |
| 2 | 1 mM | 20 µL of 0.05 M working solution + 950 µL HPLC water |
| 3 | 2.5 mM | 50 µL of 0.05 M working solution + 950 µL HPLC water |
| 4 | 5 mM | 10 µL of 0.5 M working solution + 950 µL HPLC water |
| 5 | 10 mM | 20 µL of 0.5 M working solution + 950 µL HPLC water |
| 6 | 25 mM | 50 µL of 0.5 M working solution + 950 µL HPLC water |
| 7 | 50 mM | 10 µL of stock solution + 950 µL HPLC water |

**S4 Table.** Mobile phase transition program by gradient method.

| **Time (min)** | **MF* A(%)** | **MF* B(%)** | **Flow rate(ml/min)** |
| --- | --- | --- | --- |
| 0.0 | 100 |  | 1.25 |
| 8.0 | 100 |  | 1.25 |
| 8.5 | 80 | 20 | 1.25 |
| 13.0 | 80 | 20 | 1.25 |
| 13.5 | 100 |  | 1.25 |
| 18.0 | 100 |  | 1.25 |

*MF: mobile phase

**IL6**

**S5 Table.** intergroup comparison test of IL6 along with the statistical characteristics of each group

| **P Value** | **Upper Limit** | **A-B** | **Lower Limit** | **Group B** | **Group A** |
| --- | --- | --- | --- | --- | --- |
| 4.5073e-09 | -1.3063 | -1.805 | -2.3037 | **DC** | **NDC** |
| 0.018267 | -0.079912 | -0.57862 | -1.0773 | **CET** | **NDC** |
| 0.0002922 | -0.37179 | -0.8705 | -1.3692 | **HIIT** | **NDC** |
| 1.589e-06 | 1.7251 | 1.2264 | 0.72766 | **CET** | **DC** |
| 0.00011342 | 1.4332 | 0.9345 | 0.43579 | **HIIT** | **DC** |
| 0.39604 | 0.20684 | -0.29188 | -0.79059 | **HIIT** | **CET** |
|  |  |  |  |  |  |
|  |  |  |  |  |  |
| 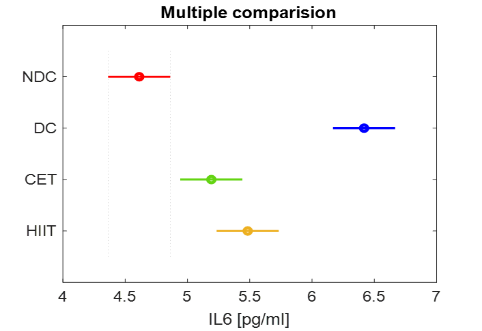 | | **SE** | **STD** | **Aver.** |  |
|  |  | 0.068899 | 0.194877 | 4.61275 | **NDC** |
|  |  | 0.093327 | 0.263969 | 6.41775 | **DC** |
|  |  | 0.152785 | 0.43214 | 5.191375 | **CET** |
|  |  | 0.172996 | 0.489306 | 5.48325 | **HIIT** |

**S6 Table.** Investigating the hypothesis of the effect of exercise interventions on sera IL6 concentration using one-way ANOVA

| **Prob>F** | **F** | **MS** | **df** | **SS** | **Source** |
| --- | --- | --- | --- | --- | --- |
| 1.77169e-09 | 34.03 | 4.54205 | 3 | 13.6262 | **Columns** |
|  |  | 0.13346 | 28 | 3.7368 | **Error** |
|  |  |  | 31 | 17.3629 | **Total** |

Note: The ANOVA table shows the between-groups variation (Columns) and within-groups variation (Error). SS is the sum of squares, and df is the degrees of freedom. The total degrees of freedom is total number of observations minus one, which is (4×8) - 1 = 31. The between-groups degrees of freedom is number of groups minus one, which is 4 - 1 = 3. The within-groups degrees of freedom is total degrees of freedom minus the between groups degrees of freedom, which is 31 - 3 = 28. MS is the mean squared error, which is SS/df for each source of variation. The F-statistic is the ratio of the mean squared errors (4.54205/0.13346). The p-value is the probability that the test statistic can take a value greater than the value of the computed test statistic, i.e., P(F > 34.03). The small p-value of 1.77169e-09 indicates that differences between column means are significant.

**TLR4**

**S7 Table.** Intergroup comparison test of TLR4 along with the statistical characteristics of each group

| **P Value** | **Upper Limit** | **A-B** | **Lower Limit** | **Group B** | **Group A** |
| --- | --- | --- | --- | --- | --- |
| 3.7817e-09 | -3.3574 | -4.4528 | -5.5481 | **DC** | **NDC** |
| 0.90244 | 0.81884 | -0.27651 | -1.3719 | **CET** | **NDC** |
| 5.9187e-05 | -1.0161 | -2.1115 | -3.2068 | **HIIT** | **NDC** |
| 3.8299e-09 | 5.2716 | 4.1763 | 3.0809 | **CET** | **DC** |
| 1.1464e-05 | 3.4366 | 2.3413 | 1.2459 | **HIIT** | **DC** |
| 0.00042026 | -0.73962 | -1.835 | -2.9303 | **HIIT** | **CET** |
|  |  |  |  |  |  |
|  |  |  |  |  |  |
| 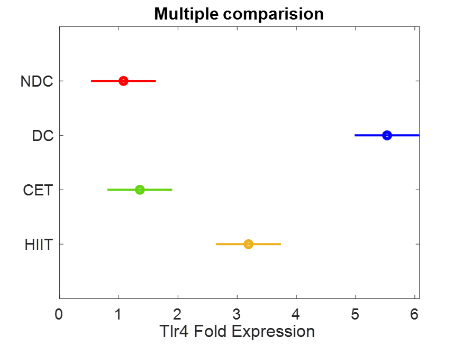 | | **SE** | **STD** | **Aver.** |  |
|  |  | 0.096138 | 0.288414 | 1.07903 | **NDC** |
|  |  | 0.419025 | 1.257076 | 5.531801 | **DC** |
|  |  | 0.214447 | 0.64334 | 1.355543 | **CET** |
|  |  | 0.309969 | 0.929908 | 3.190517 | **HIIT** |

**S8 Table.** Investigating the hypothesis of the effect of exercise interventions on cecal TLR4 expression using one-way ANOVA

| **Prob>F** | **F** | **MS** | **df** | **SS** | **Source** |
| --- | --- | --- | --- | --- | --- |
| 2.29694e-12 | 51.65 | 37.9889 | 3 | 113.967 | Columns |
|  |  | 0.7355 | 32 | 23.536 | Error |
|  |  |  | 35 | 137.503 | Total |

***Akkermansia muciniphila***

**S9 Table.** Intergroup comparison test of *Akkermansia* along with the statistical characteristics of each group

| **P Value** | **Upper Limit** | **A-B** | **Lower Limit** | **Group B** | **Group A** |
| --- | --- | --- | --- | --- | --- |
| 1.0054e-06 | 0.95974 | 0.74974 | 0.53973 | **DC** | **NDC** |
| 2.826e-05 | 0.75546 | 0.54545 | 0.33544 | **CET** | **NDC** |
| 0.11068 | 0.38676 | 0.17675 | -0.03326 | **HIIT** | **NDC** |
| 0.057475 | 0.0057246 | -0.20429 | -0.41429 | **CET** | **DC** |
| 1.7161e-05 | -0.3629 | -0.57298 | -0.78299 | **HIIT** | **DC** |
| 0.0010756 | -0.15869 | -0.3687 | -0.57871 | **HIIT** | **CET** |
|  |  |  |  |  |  |
|  |  |  |  |  |  |
|  | | **SE** | **STD** | **Aver.** |  |
|  |  | 0.053093 | 0.106187 | 1.36675 | **NDC** |
|  |  | 0.043955 | 0.08791 | 0.617015 | **DC** |
|  |  | 0.039239 | 0.078478 | 0.8213 | **CET** |
|  |  | 0.060964 | 0.121929 | 1.19 | **HIIT** |

**S10 Table.** Investigating the hypothesis of the effect of exercise interventions on cecal *Akkermansia* expression using one-way ANOVA

| **Prob>F** | **F** | **MS** | **df** | **SS** | **Source** |
| --- | --- | --- | --- | --- | --- |
| 6.96924e-07 | 46.53 | 0.46561 | 3 | 1.39684 | **Columns** |
|  |  | 0.01001 | 12 | 0.12009 | **Error** |
|  |  |  | 15 | 1.51693 | **Total** |

***Butyrivibrio fibrisolvens***

**S11 Table.** Intergroup comparison test of *Butyrivibrio* along with the statistical characteristics of each group

| **P Value** | **Upper Limit** | **A-B** | **Lower Limit** | **Group B** | **Group A** |
| --- | --- | --- | --- | --- | --- |
| 0.00011803 | 1.9881 | 1.3754 | 0.76263 | **DC** | **NDC** |
| 0.00067672 | 1.7484 | 1.1357 | 0.52292 | **CET** | **NDC** |
| 0.1415 | 1.0972 | 0.48443 | -0.12831 | **HIIT** | **NDC** |
| 0.66078 | 0.37304 | -0.23971 | -0.85246 | **CET** | **DC** |
| 0.0047828 | -0.27819 | -0.89094 | -1.5037 | **HIIT** | **DC** |
| 0.036179 | -0.038483 | -0.65123 | -1.264 | **HIIT** | **CET** |
|  |  |  |  |  |  |
|  |  |  |  |  |  |
|  | | **SE** | **STD** | **Aver.** |  |
|  |  | 0.110057 | 0.220115 | 1.790975 | **NDC** |
|  |  | 0.029008 | 0.058016 | 0.415541 | **DC** |
|  |  | 0.129952 | 0.259904 | 0.65525 | **CET** |
|  |  | 0.235267 | 0.470535 | 1.30648 | **HIIT** |

**S12 Table.** Investigating the hypothesis of the effect of exercise interventions on cecal *Butyrivibrio* expression using one-way ANOVA

| **Prob>F** | **F** | **MS** | **df** | **SS** | **Source** |
| --- | --- | --- | --- | --- | --- |
| 8.85221e-05 | 18.36 | 1.5638 | 3 | 4.6914 | **Columns** |
|  |  | 0.08519 | 12 | 1.02231 | **Error** |
|  |  |  | 15 | 5.71371 | **Total** |

***prevotella copri***

**S13 Table.** Intergroup comparison test of *Prevotella* along with the statistical characteristics of each group

| **P Value** | **Upper Limit** | **A-B** | **Lower Limit** | **Group B** | **Group A** |
| --- | --- | --- | --- | --- | --- |
| 0.00016535 | -1.8307 | -2.9984 | -4.1662 | **DC** | **NDC** |
| 0.0059555 | -0.58067 | -1.7484 | -2.9162 | **CET** | **NDC** |
| 0.98271 | 1.036 | -0.13177 | -1.2995 | **HIIT** | **NDC** |
| 0.036456 | 2.4178 | 1.25 | 0.082225 | **CET** | **DC** |
| 0.00022793 | 4.0344 | 2.8667 | 1.6989 | **HIIT** | **DC** |
| 0.0094158 | 2.7844 | 1.6167 | 0.44889 | **HIIT** | **CET** |
|  |  |  |  |  |  |
|  |  |  |  |  |  |
|  | | **SE** | **STD** | **Aver.** |  |
|  |  | 0.091351 | 0.158224 | 0.511559 | **NDC** |
|  |  | 0.418848 | 0.725465 | 3.51 | **DC** |
|  |  | 0.172143 | 0.298161 | 2.26 | **CET** |
|  |  | 0.229226 | 0.397031 | 0.643333 | **HIIT** |

**S14 Table.** Investigating the hypothesis of the effect of exercise interventions on cecal *Prevotella* expression using one-way ANOVA

| **Prob>F** | **F** | **MS** | **df** | **SS** | **Source** |
| --- | --- | --- | --- | --- | --- |
| 9.76498e-05 | 30.66 | 6.11474 | 3 | 18.3442 | **Columns** |
|  |  | 0.19947 | 8 | 1.5957 | **Error** |
|  |  |  | 11 | 19.9399 | **Total** |

**Butyrate**

**S15 Table.** Intergroup comparison test of *butyrate* along with the statistical characteristics of each group

| **P Value** | **Upper Limit** | **A-B** | **Lower Limit** | **Group B** | **Group A** |
| --- | --- | --- | --- | --- | --- |
| 0.01077 | 8.1281 | 4.6723 | 1.2166 | **DC** | **NDC** |
| 0.033717 | 7.2154 | 3.7597 | 0.30389 | **CET** | **NDC** |
| 0.80549 | 4.4268 | 0.971 | -2.4848 | **HIIT** | **NDC** |
| 0.83165 | 2.5431 | -0.91267 | -4.3684 | **CET** | **DC** |
| 0.036351 | -0.24556 | -3.7013 | -7.1571 | **HIIT** | **DC** |
| 0.11952 | 0.66711 | -2.7887 | -6.2444 | **HIIT** | **CET** |
|  |  |  |  |  |  |
|  |  |  |  |  |  |
| 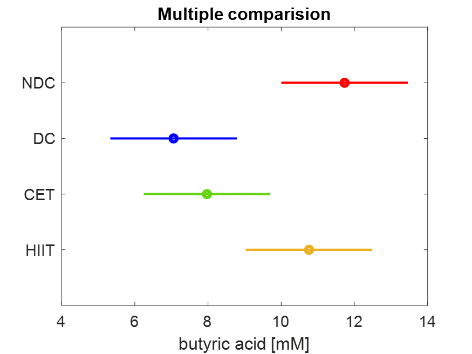 | | **SE** | **STD** | **Aver.** |  |
|  |  | 1.031006 | 1.785756 | 11.731 | **NDC** |
|  |  | 0.258752 | 0.448171 | 7.058667 | **DC** |
|  |  | 0.214732 | 0.371927 | 7.971333 | **CET** |
|  |  | 1.073794 | 1.859866 | 10.76 | **HIIT** |

**S16 Table.** Investigating the hypothesis of the effect of exercise interventions on cecal *butyrate* concentration using one-way ANOVA

| **Prob>F** | **F** | **MS** | **df** | **SS** | **Source** |
| --- | --- | --- | --- | --- | --- |
| 0.0073 | 8.48 | 14.8045 | 3 | 44.4136 | Columns |
|  |  | 1.7468 | 8 | 13.9744 | Error |
|  |  |  | 11 | 58.388 | Total |

**propionate**

**S17 Table.** Intergroup comparison test of *propionate* along with the statistical characteristics of each group

| **P Value** | **Upper Limit** | **A-B** | **Lower Limit** | **Group B** | **Group A** |
| --- | --- | --- | --- | --- | --- |
| 0.0022492 | 0.38275 | 0.24367 | 0.10459 | **DC** | **NDC** |
| 0.0088414 | 0.33375 | 0.19467 | 0.055586 | **CET** | **NDC** |
| 0.76316 | 0.18175 | 0.042667 | -0.96414 | **HIIT** | **NDC** |
| 0.68377 | 0.09008 | -0.049 | -0.18808 | **CET** | **DC** |
| 0.0073412 | -0.06192 | -0.201 | -0.34008 | **HIIT** | **DC** |
| 0.032981 | -0.01292 | -0.152 | -0.29108 | **HIIT** | **CET** |
|  |  |  |  |  |  |
|  |  |  |  |  |  |
|  | | **SE** | **STD** | **Aver.** |  |
|  |  | 0.025899 | 0.044859 | 0.258333 | **NDC** |
|  |  | 0.002906 | 0.005033 | 0.014667 | **DC** |
|  |  | 0.03874 | 0.067099 | 0.063667 | **CET** |
|  |  | 0.039905 | 0.069118 | 0.215667 | **HIIT** |

**S18 Table.** Investigating the hypothesis of the effect of exercise interventions on cecal *propionate* concentration using one-way ANOVA

| **Prob>F** | **F** | **MS** | **df** | **SS** | **Source** |
| --- | --- | --- | --- | --- | --- |
| 0.0013 | 14.58 | 0.04125 | 3 | 0.12375 | **Columns** |
|  |  | 0.00283 | 8 | 0.02263 | **Error** |
|  |  |  | 11 | 0.14638 | **Total** |

­
